# A Rapid Route to Aminocyclopropanes via Carbamatoorganozinc Carbenoids**

**DOI:** 10.1002/anie.201304720

**Published:** 2013-08-01

**Authors:** Shingo Ishikawa, Tom D Sheppard, Jarryl M D'Oyley, Akio Kamimura, William B Motherwell

**Affiliations:** Department of Chemistry, University College London20 Gordon St, London, WC1H 0AJ (UK); Department of Applied Molecular Bioscience, Graduate School of Medicine, Yamaguchi UniversityUbe 755-8611 (Japan)

**Keywords:** carbenoids, copper, small ring systems, synthetic methods, zinc

The aminocyclopropane unit can be found in a wide variety of biologically active natural products and pharmaceuticals,^1^ and its inherent reactivity can be harnessed in synthetically useful ring-opening reactions.^2^ The preparation of aminocyclopropanes has attracted considerable attention, though many approaches require functional-group manipulation of a preformed cyclopropane, as in the Curtius rearrangement of cyclopropylcarboxylic acids,^3^ reduction of nitrocyclopropanes,^4^ reductive amination of cyclopropanone derivatives,^5^ and the reaction of cyclopropylchloroboranes with azides.^6^ The cyclopropanation of geometrically defined enamine and enamide derivatives^7^ and the elegant variants of the Kulinkovich reaction^8^ using amides^9^ or nitriles,^10^ also provide versatile methods. Conceptually, the simplest approach would be the addition of a protected aminocarbenoid to an alkene, as this would enable the preparation of aminocyclopropanes from the vast array of readily accessible geometrically defined alkenes. Unfortunately, save for a beautiful example by Barluenga et al. involving a chromium-based dialkylamino carbenoid,^11^ Fischer carbenoids cannot be used in such reactions since they undergo competing metathesis. Herein, we report a simple and direct one pot synthesis of protected aminocyclopropanes, a reaction which features the first in situ generation of hitherto unknown carbamatoorganozinc carbenoids (Scheme 1).

**Scheme 1:**
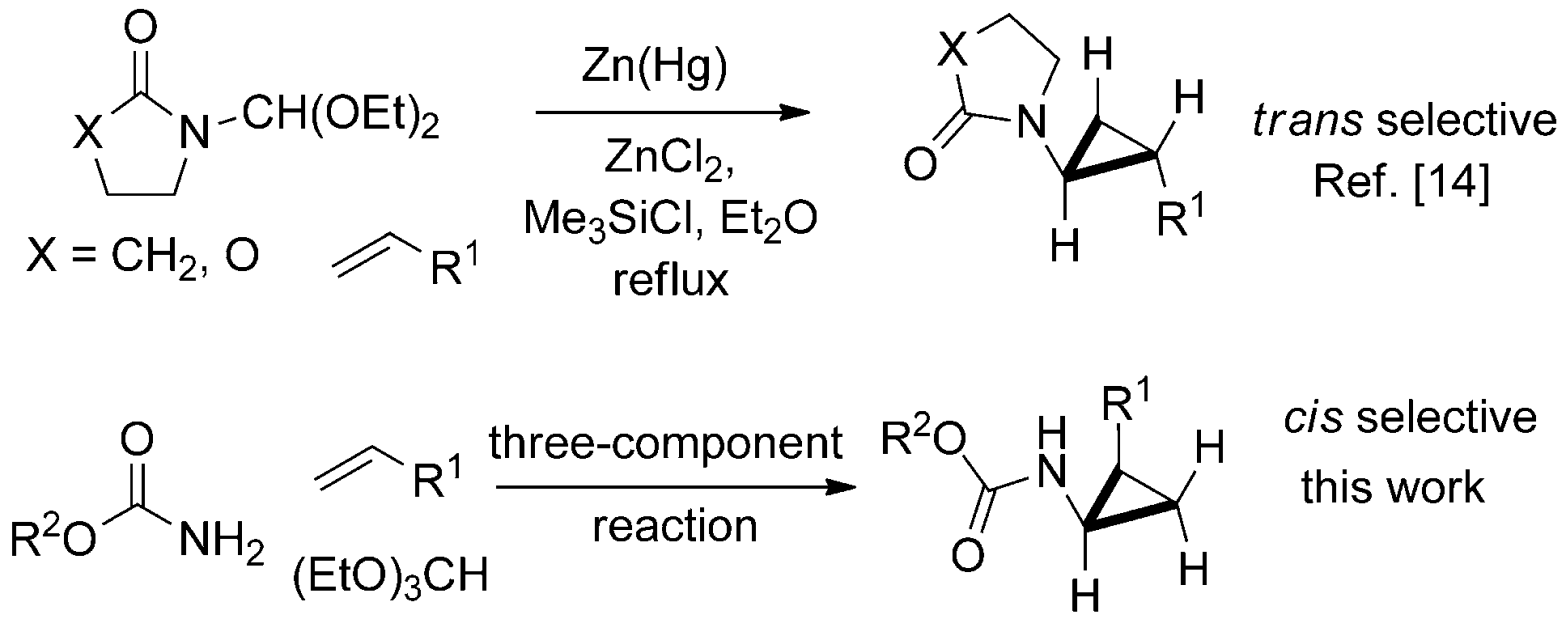
Zinc-mediated amidocyclopropanation and direct carbamatocyclopropanation of alkenes.

We have previously shown that functionalized organozinc carbenoids can be generated from carbonyl compounds or acetals by reductive deoxygenation with zinc in the presence of Me_3_SiCl,^12^ and this strategy can be extended to the generation of heteroatom-substituted carbenoids using orthoesters (alkoxycyclopropanation)^13^ or *N*-diethoxymethyllactams (amidocyclopropanation).^14^ In the latter case, related chiral diphenyloxazolidinone carbenoid precursors^15^ can provide access to free aminocyclopropanes after hydrogenolytic deprotection in certain cases. In spite of the above observations, the search for a simple and direct reaction for delivering a usefully protected aminocyclopropane had remained frustratingly elusive. As a consequence of the commercial importance of such compounds as Tranylcypromine, the subset of aminocyclopropanes which also bear an aromatic substituent on the adjacent carbon atom is of particular interest. As emphasized by de Meijere et al.,^16^ any sequence which involves liberation of the free amine by a deprotection step using hydrogenolysis, also leads to concomitant ring opening of the benzylic cyclopropane for this class of compounds, and this problem has also been unwittingly encountered by others.^17^

In light of the above situation, we therefore elected to attempt the sequence outlined in Scheme 2 involving conversion of a simple carbamate into the diethoxymethyl derivative **1**, as a precursor for subsequent evolution of the putative carbenoid **2** on reduction with zinc in the presence of Me_3_SiCl.

**Scheme 2:**
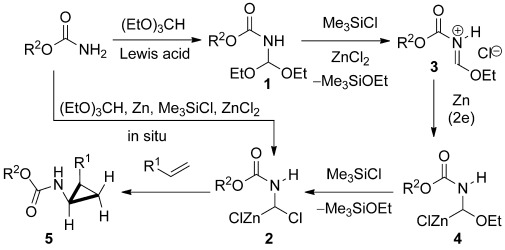
Proposed carbamatocyclopropanation.

A plausible series of intermediates is shown, and requires two-electron delivery from zinc to the cation **3**, generated by Lewis acid assisted cleavage of one of the ethoxy groups in **1**. Further reaction of **4** with Me_3_SiCl then furnishes the carbenoid **2** which can be trapped by an alkene to give the carbamatocyclopropane **5**. In the event, all efforts to prepare and isolate the desired precursor **1** were uniformly unsuccessful,^18^ with the NMR spectra of the complex reaction mixtures indicating the presence, inter alia, of the *N*,*N*-bis(diethoxymethyl) derivative and the imino ether. Undeterred by these observations however, and in spite of the fact that competing alkoxycyclopropanation^13^ or further reaction of the product leading to double cyclopropanation could take place, we decided to explore an even more adventurous one-pot sequence involving in situ formation of the carbenoid **2**.

Fortunately, it was possible to take advantage of ongoing contemporaneous work, both on the optimization of the amidocyclopropanation reaction using the preformed carbenoid precursor **8**, as well as the development of an in situ protocol using the oxazolidinone **9** and (EtO)_3_CH (Table 1). As in the case of its classical counterpart, the Simmons–Smith reaction,^19^ a particular aspect of interest lay in the selection of the metallic reducing agent given the heterogeneous nature of the reaction conditions and the often employed tactic of using zinc-copper couples or zinc amalgam. The surprising results for a comparative study involving metallic zinc, in situ generation of a zinc-copper couple, and the very unusual alternative of using both zinc and copper metals simultaneously are presented in Table 1. To the best of our knowledge, this latter combination which does not involve prior formation of an alloy, an amalgam, or a couple, is without precedent.

**Table 1:** Optimization of Zn/Cu mediated cyclopropanation reactions
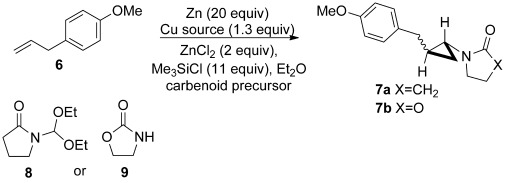

Entry	Carbenoid Precursor	Cu source	*T* [h]	Yield [%]
1	**8** (2 equiv)	none	16	30[a]
2	**8** (2 equiv)	CuCl	16	63[a]
3	**8** (2 equiv)	CuCl_2_	16	75[a]
4	**8** (2 equiv)	Cu (s)	16	92[a]
5	**8** (2 equiv)	Cu[b]	16	0[a]
6	**9** (3 equiv)+(EtO)_3_CH (3.6 equiv)	none	72	51[c]
7	**9** (3 equiv)+(EtO)_3_CH (3.6 equiv)	CuCl	72	38[c]
8	**9** (3 equiv)+(EtO)_3_CH (3.6 equiv)	CuCl_2_	72	44[c]
9	**9** (3 equiv)+(EtO)_3_CH (3.6 equiv)	Cu(s)	72	54[c]

[a] Reaction carried out at reflux.

[b] Cu (20 equiv); no Zn used.

[c] Reaction carried out at room temperature.

Thus, as clearly revealed in entries 1–5 of Table 1, the combination of the two metals (entry 4) leads to a very dramatic improvement over the use of either zinc alone (entry 1), or in situ generated zinc-copper couples (entries 2 and 3), and, as expected, no reaction occurs in the presence of copper alone (entry 5). Curiously, for the one-pot cyclopropanation method using the oxazolidinone **9**, this effect is less marked (entries 6–9), although on the basis of experience, inclusion of copper metal tends to lead to more consistent results. Whilst it is tempting to speculate on the possible evolution of some form of organocoppper or heterobimetallic carbenoid species during these reactions, the origin and substrate dependence of this “decoupling” protocol remain unresolved at present.

To our delight, the result of applying the optimal reaction conditions to direct carbamatocyclopropanation of **6** using MeOCONH_2_ and (EtO)_3_CH afforded the cyclopropane **10** in 78 % yield (Scheme 3), thus effectively mirroring those described above for **9**, save for the fact that the reactions were even faster at room temperature. With this mild and convenient method in hand, the scope of the reaction was then explored with respect to the alkene component.

**Scheme 3:**
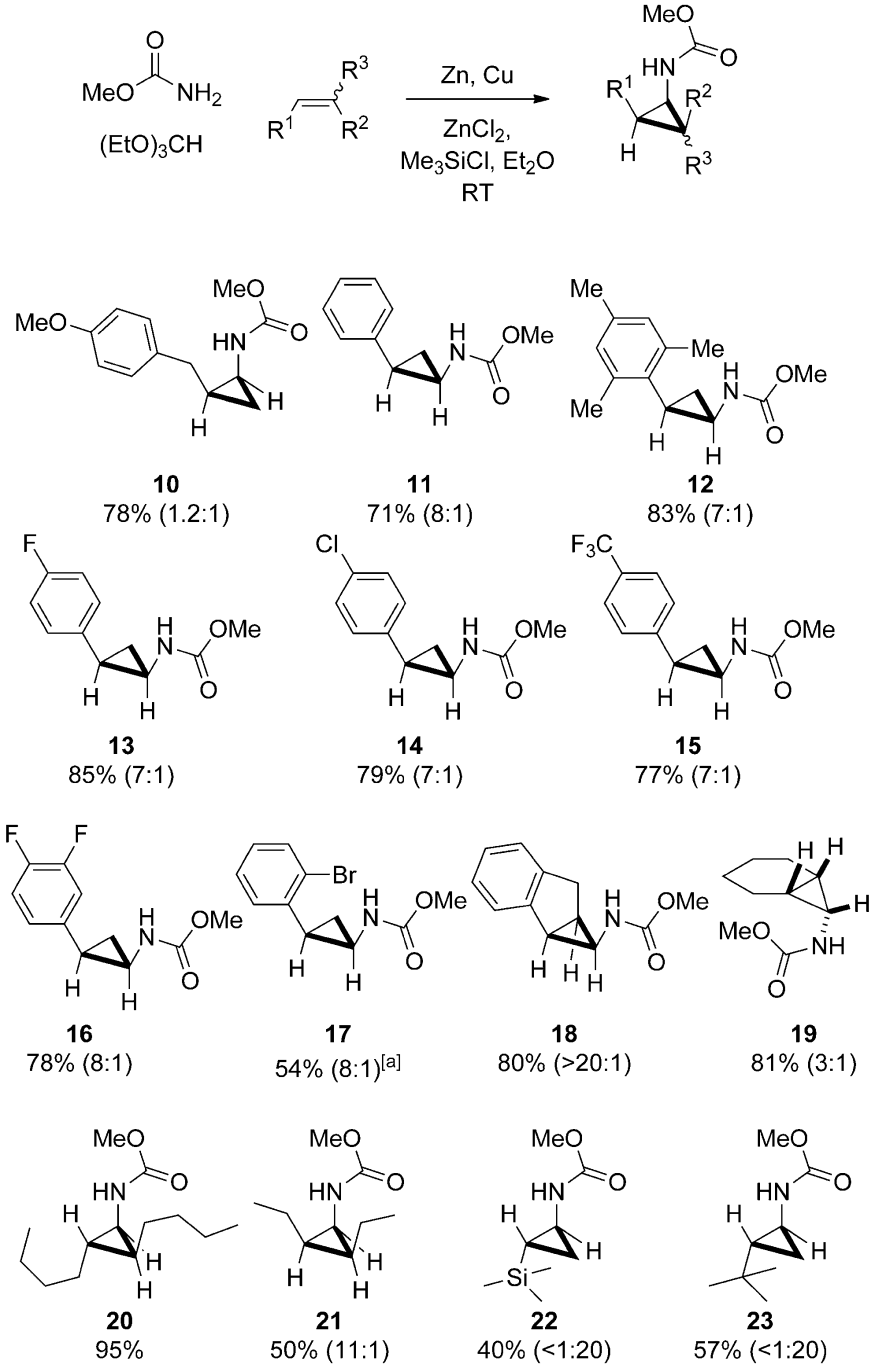
Cyclopropanation reactions with methyl carbamate. Major diastereoisomers are shown, with the d.r. value within parentheses (*cis*/*trans* or *endo*/*exo*). [a] A solution of alkene in (EtO)_3_CH was added dropwise to the other reagents.

Examination of the results encapsulated in Scheme 3 reveals that the desired cyclopropanes can be formed smoothly and in preparatively useful yields, especially for the important subset of styrene derivatives **11**–**18**. In contrast to the classical Simmons–Smith reaction, it was also gratifying that only 3.0 and 3.5 equivalents of MeOCONH_2_and (EtO)_3_CH, respectively, are required. In direct contrast to the behavior exhibited by the organozinc carbenoids derived from the precursors **8** and **9**, the present carbamatocyclopropanation reaction displays an unusually high preference for formation of the more hindered *cis* or *endo* diastereoisomer.^14^, ^15^ Typical selectivities for the family of styrene derivatives **11**–**17** were 7–8:1. Notably, there are very few reported *cis*-selective cyclopropanation reactions employing functionalized carbenoids.^20^ The cyclic alkenes indene and cyclohexene also gave the corresponding *endo* cyclopropanes **18** and **19**, respectively, with excellent and moderate stereoselectivity. Even though the expected retention of alkene geometry was observed in the formation of **20** and **21**, the formation of the all-*cis*-cyclopropane **21** with excellent stereoselectivity (11:1) is particularly noteworthy. The only exceptions to this *cis*/*endo* preference can be found in the reactions of alkenes with the very bulky Me_3_Si and *t*Bu groups which favored exclusive formation of the *trans* isomers **22** and **23**, respectively, and the reaction of *p*-allyl anisole providing an almost equimolar ratio of the two diastereoisomers of **10**.

From a practical viewpoint it should also be noted that the crystalline nature of the methyl-carbamate-protected cyclopropanes facilitates purification on a larger scale. Thus, cyclopropanation of styrene on a 5 gram scale gave the pure *cis* cyclopropane **11** in 47 % yield after a single recrystallization (Scheme 4).

**Scheme 4:**
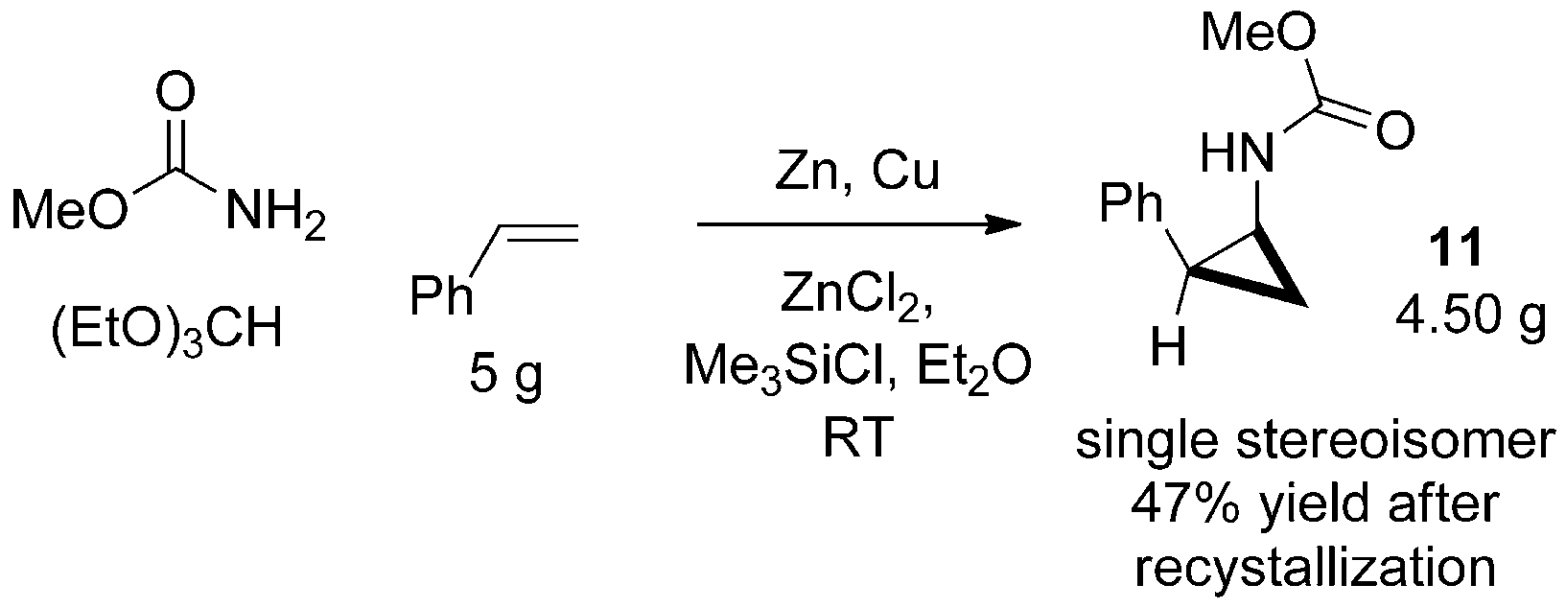
Gram-scale synthesis of the cyclopropane **11**.

A plausible rationale for the observed sterechemical preference of this reaction is shown in Figure 1. In contrast to the spatial requirements of the geometrically constrained lactam- and oxazolidinone-derived carbenoids explored previously,^14^, ^15^ the unhindered carbamato group has many more degrees of conformational freedom. Thus, it may be possible to adopt a conformation in which the approach of the alkene is dominated by a preference to orient the substituent on the alkene as far away as possible from the bulky zinc carbenoid center.

**Figure 1:**
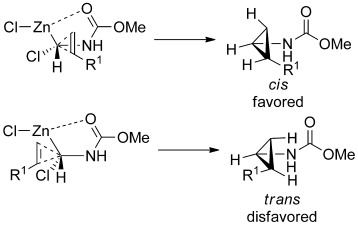
Possible transition states for the *cis* and *trans* carbamatocyclopropanation reactions.

Our attention was then directed towards the final deprotection step (Scheme 5). Pleasingly, treatment of representative examples of the cyclopropyl methyl carbamates with iodotrimethylsilane in chloroform and subsequent addition of methanol enabled the isolation of the corresponding aminocyclopropanes as their crystalline HI salts in excellent yield. The efficiency of the overall sequence can be gauged from the fact that the cyclopropane **26**, the *cis* isomer of the AstraZeneca drug candidate AZD6140,^21^ can be obtained in only two steps and 62 % overall yield.

**Scheme 5:**
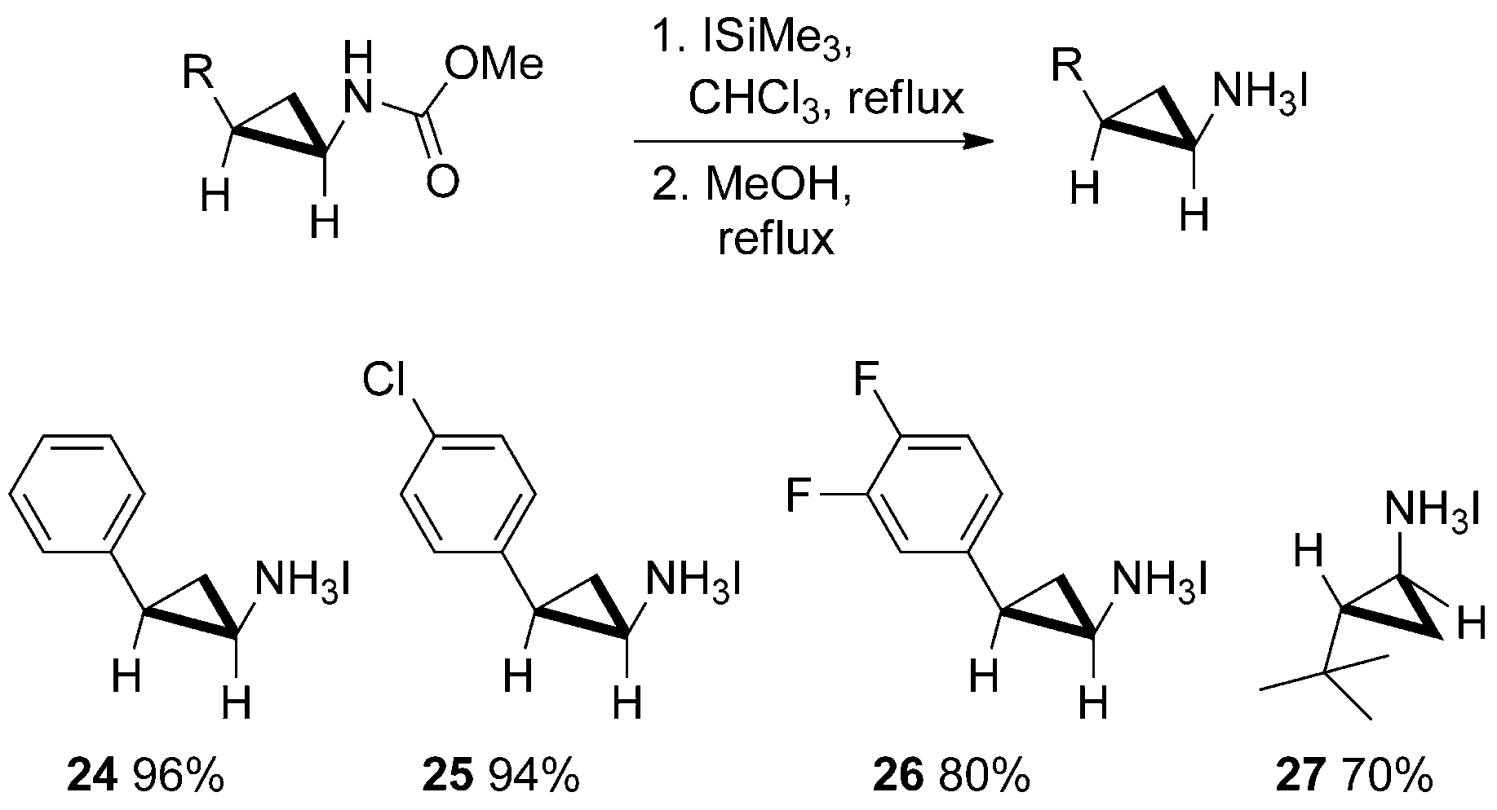
Synthesis of aminocyclopropane HI salts.

Finally, we have also briefly examined the extension of the reaction to the use of BnOCONH_2_ (Scheme 6), thus highlighting the fact that a complementary protecting group is also available for those aminocyclopropanes which are not prone to hydrogenolysis.

**Scheme 6:**
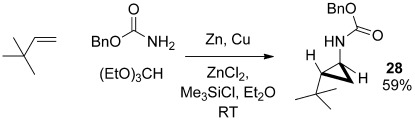
Synthesis of a benzyl-carbamate-protected cyclopropane.

In summary, we have developed a simple, practical, and very inexpensive method for the preparation of carbamate-protected aminocyclopropanes that proceeds via hitherto unknown carbamatoorganozinc carbenoids. Reactions proceed smoothly under mild reaction conditions at room temperature and with a preference for the formation of the *cis*/*endo* isomer. Deprotection to give the HI salts of the free amines is readily achieved using iodotrimethylsilane.

## Experimental Section

Triethyl orthoformate (1.2 mL, 7.20 mmol) was added dropwise by syringe pump (1.0 mL h^−1^) to a vigorously stirred mixture of MeOCONH_2_ (463 mg, 6.17 mmol), Zn (2.65 g, 40.46 mmol), Cu (175 mg, 2.75 mmol), ZnCl_2_ (828 mg, 6.07 mmol), Me_3_SiCl (2.8 mL, 22.00 mmol), and alkene (1.94 mmol) in anhydrous Et_2_O (10 mL) under nitrogen. The reaction mixture was stirred at room temperature for 3–16 h. After quenching with sat. aq. NaHCO_3_ (10 mL), the resulting suspension was filtered and the solid was washed with Et_2_O (2×25 mL). The biphasic mixture was extracted with Et_2_O (3×30 mL) and the combined organic layers were washed with brine (10 mL), dried over MgSO_4_, filtered and concentrated.

Potassium carbonate (830 mg, 6.00 mmol)^22^ was added to the solution of the residue in MeOH (2 mL). After stirring for 1 h, the reaction mixture was concentrated in vacuo and water (10 mL) was added to the residue. The aqueous layer was extracted with CH_2_Cl_2_ (3×30 mL). The organic layer was washed with brine (10 mL), dried over MgSO_4_, filtered, and concentrated in vacuo. The crude reaction mixture was purified by flash column chromatography to give the carbamatoocyclopropane.

Iodotrimethylsilane (0.28 mL, 2.0 mmol) was added to a solution of the amidocyclopropane (202 mg, 1.06 mmol) in CHCl_3_ (10 mL). The reaction mixture was stirred at reflux for 1 hour. After cooling to room temperature, MeOH (2 mL) was added to the reaction mixture and it was stirred at reflux for 30 min. The reaction mixture was concentrated and the resultant solid was washed with Et_2_O to give the aminocyclopropane hydrogen iodide salt.

## Supplementary material

As a service to our authors and readers, this journal provides supporting information supplied by the authors. Such materials are peer reviewed and may be re-organized for online delivery, but are not copy-edited or typeset. Technical support issues arising from supporting information (other than missing files) should be addressed to the authors.
