# Supplementary material for: A Rapid Route to Aminocyclopropanes via Carbamatoorganozinc Carbenoids
Source: Angew Chem Int Ed Engl. 2013 Aug 1;52(38):10060–3. doi: 10.1002/anie.201304720 (PMC4065353; doi:10.1002/anie.201304720)
Supplement: Supplementary file 1 [file anie0052-10060-SD1.pdf]

Angewandte Chemie
Eine Zeitschrift der Gesellschaft Deutscher Chemiker

Supporting Information
© Wiley-VCH 2013
69451 Weinheim, Germany

A Rapid Route to Aminocyclopropanes via Carbamatoorganozinc Carbenoids**

*Shingo Ishikawa, Tom D. Sheppard, Jarryl M. D'Oyley, Akio Kamimura, and William B. Motherwell**

anie_201304720_sm_miscellaneous_information.pdf

Contents

1. General Experimental Procedures 2
2. Spectroscopic Data for all new Compounds 3
3. ^{1}H and ^{13}C NMR Spectra for all new Compounds 13

General Experimental Procedures

All reactions were carried out in oven-dried glassware under a nitrogen atmosphere unless otherwise indicated. Diethyl ether was used following purification from an anhydrous engineering zeolite drying apparatus. Chlorotrimethylsilane was distilled from calcium hydride immediately prior to use. All alkenes purchased from commercial suppliers or prepared via literature procedures.[1] Column chromatography was carried out using BDH (40-60 µm) silica gel and analytical thin layer chromatography was carried out using Merck Keiselgel aluminium-backed plates coated with silica gel. Components were visualised using combinations of ultra-violet lights, iodine, ceric ammonium molybdate, phosphomolybdic acid and potassium permanganate. Melting points were determined using a Reichert hot-stage apparatus and are uncorrected. Infrared (IR) spectra were recorded on a Perkin-Elmer 1605 Fourier transform spectrometer as thin films. ^{1}H NMR spectra were recorded at 400 MHz on a Avance 400 spectrometer, at 500 MHz on a Bruker Avance 500 spectrometer, or at 600 MHz on a Bruker Avance 600 spectrometer in the stated solvent using residual protic solvent $CHCl_3$ (δ = 7.26 ppm, s), DMSO (δ = 2.56 ppm, qn) or D_2O (4.79, s) as the internal standard. Chemical shifts are quoted in ppm using the following abbreviations: s, singlet; d, doublet; t, triplet; q, quartet; m, multiplet; br, broad or a combination of these. ^{13}C NMR spectra were recorded at 100 MHz on a Avance 400 spectrometer, at 125 MHz on a Bruker Avance 500 spectrometer or at 150 MHz on a Bruker Avance 600 spectrometer in the stated solvent using the central reference of $CHCl_3$ (δ = 77.0 ppm, t), as the internal standard. Chemical shifts are reported to the nearest 0.1 ppm. The ^{1}H and ^{13}C NMR of the carbamate protected aminocyclopropanes were run at 330 K to aid resolution of the rotameric mixtures. Mass spectra were performed at the Department of Chemistry, University College London or by the EPRSC National Mass Spectrometry Facility.

Stereochemistry of Cyclopropane Products

The relative stereochemistry of the cyclopropane isomers was assigned on the basis of ^{1}H NMR coupling constants in accordance with our previous reports.[2] The diasteresomeric ratios quoted in the manuscript were determined from the crude ^{1}H NMR spectra. The ratio of stereoisomers obtained after chormatograpic purification is given below for each compound. Where possible, ^{1}H and/or ^{13}C NMR signals for both isomers are reported below.

***trans*-1-[2-(4-Methoxybenzyl)cyclopropyl)]pyrrolidin-2-one 7a**

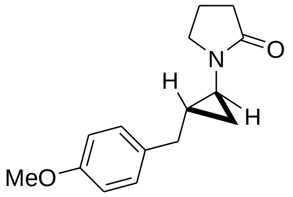

92% Yield, colourless oil; IR (neat): ν_{max} 1674, 1511 cm^{-1}; ^{1}H NMR (*trans*, 500 MHz, $CDCl_3$): δ 7.14 (d, J = 8.6 Hz, 2 H), 6.82 (d, J = 8.6 Hz, 2 H), 3.77 (s, 3 H), 3.20 (t, *J* = 7.1 Hz, 2 H), 2.65 (dd, *J* = 14.7, 6.7 Hz, 1 H), 2.52 (dt, *J* = 7.6, 3.8 Hz, 1 H), 2.49 (dd, *J* = 14.7, 7.2 Hz, 1 H), 2.43 (t, *J* = 8.1 Hz, 2 H), 1.96-1.89 (m, 2 H), 1.27 (ddddd, *J* = 9.4, 7.0, 6.8, 6.0, 3.6 Hz, 1 H), 1.21-1.12 (m, 1 H), 0.94 (ddd, *J* = 9.4, 5.8, 4.1 Hz, 1 H), 0.73 (br q, *J* = 6.5 Hz, 1 H); ^{13}C NMR (*trans*, 125 MHz, $CDCl_3$): δ 175.6, 158.2, 132.7, 129.3, 113.9, 55.2, 47.2, 37.3, 31.9, 31.7, 19.3, 18.0, 12.0; HRMS: *m/z* $[MH]^+$ calcd for $C_{15}H_{20}NO_2$: 246.14940; found: 246.14864.

trans-3-[2-(4-Methoxybenzyl)cyclopropyl)]oxazolidin-2-one 7b

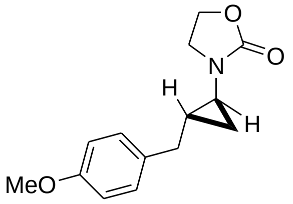

54% Yield, colourless oil; IR (neat): ν_{max} 1744, 1511 cm^{-1}; ^{1}H NMR (*trans*, 500 MHz, $CDCl_3$): δ 7.14 (d, *J* = 8.7 Hz, 2 H), 6.82 (d, *J* = 8.7 Hz, 2 H), 4.20 (dd, *J* = 8.4, 4.2 Hz, 1 H), 4.19 (dd, *J* = 8.4, 4.4 Hz, 1 H), 3.78 (s, 3 H), 3.42-3.35 (m, 2 H), 2.63 (dd, *J* = 14.7, 6.9 Hz, 1 H), 2.52 (dd, *J* = 14.7, 7.0 Hz, 1 H), 2.35 (dt, *J* = 7.0, 3.5 Hz, 1 H), 1.30 (ddddd, *J* = 9.8, 7.0, 6.8, 5.9, 3.2 Hz, 1 H), 0.99 (ddd, *J* = 9.4, 5.8, 3.8 Hz, 1 H), 0.75 (br q, *J* = 6.4 Hz, 1 H); ^{13}C NMR (*trans*, 125 MHz, $CDCl_3$): δ 158.4, 157.9, 132.4, 129.2, 113.7, 61.7, 55.1, 45.6, 36.8, 31.9, 20.4, 12.8; HRMS: *m/z* $[MH]^+$ calcd for $C_{14}H_{18}NO_3$: 248.12866; found: 248.12892.

Methyl *trans*-2-(4-methoxybenzyl)cyclopropylcarbamate 10

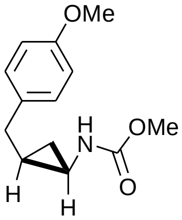

Yield 78%, (*cis:trans*=1.2:1); IR (neat): ν_{max} 3268, 1710, 1683, 1548, 1510, 1446 cm^{-1}; ^{1}H NMR (*cis*, 400MHz, 330K, $CDCl_3$): δ 7.15 (d, *J* = 8.1 Hz, 2H), 6.83 (d, *J* = 8.3 Hz, 2H), 4.94 (br, 1 H), 3.77 (s, 3 H), 3.66 (s, 3 H), 2.79-2.71 (m, 2 H), 2.52 (dd, *J* = 15.2, 7.9 Hz, 1 H), 1.26-1.08 (m, 1 H), 0.99 (td, *J* = 8.1, 5.9 Hz, 1 H), 0.36 (br q, *J* = 5.8 Hz, 1 H); ^{13}C NMR (*cis*, 101 MHz, $CDCl_3$): δ 158.0, 157.9, 133.3, 128.9, 114.0, 55.1, 51.8, 32.5, 27.8, 18.2, 12.4; ^{1}H NMR (*trans*, 400MHz, 330K, $CDCl_3$): δ 7.13 (d, *J* = 8.5 Hz, 2H), 6.82 (d, *J* = 8.5 Hz, 2H), 4.99 (br, 1 H), 3.77 (s, 3 H), 3.63 (s, 3 H), 2.67 (dd, *J* = 14.7, 6.4 Hz, 1 H), 2.49-2.42 (m, 2 H), 1.26-1.08 (m, 1 H), 0.72 (ddd, *J* = 9.1, 5.5, 3.8 Hz, 1 H), 0.64 (br q, *J* = 6.3 Hz, 1 H); ^{13}C NMR (*trans*, 101 MHz, $CDCl_3$): δ 158.1, 157.5, 132.7, 129.2, 113.8, 55.1, 51.7, 36.9, 29.7, 21.5, 13.5; HRMS: *m/z* [MH^+] calcd for $C_{13}H_{18}NO_3$: 236.12867; found: 236.12878.

Methyl *cis*-2-phenylcyclopropylcarbamate 11

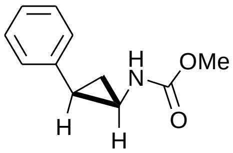

71% Yield (*cis*:*trans*, 8:1); white crystals, mp 68.5-68.6 °C (hexane-EtOAc); IR (neat): ν_{max} 3346, 3025, 3003, 2971, 1738, 1682 cm^{-1}; ^{1}H NMR (*cis*, 400 MHz, 330 K, $CDCl_3$): δ 7.62-6.99 (m, 5 H), 4.31 (br, 1 H), 3.61 (s, 3 H), 3.03-2.96 (m, 1 H), 2.29 (br q, *J* = 8.0 Hz, 1 H), 1.34 (ddd, *J* = 9.0, 7.0, 6.4 Hz, 1 H), 1.08-1.03 (m, 1 H); ^{13}C NMR (*cis*, 126 MHz, $CDCl_3$): δ 157.5, 136.1, 128.7, 128.2, 126.4, 51.9, 29.0, 21.7, 12.0. ^{1}H NMR (*trans*, 400 MHz, 330 K, $CDCl_3$): δ 7.69-6.75 (m, 5 H), 4.88 (br, 1 H), 3.71 (s, 3 H), 2.77 (dddd, *J* = 7.4, 4.3, 3.3, 2.3 Hz, 1 H), 2.09 (ddd, *J* = 9.7, 6.4, 3.3 Hz, 1 H), 1.21 (dt, *J* = 7.3, 6.1 Hz, 1 H), 1.18-1.12 (m, 1 H); ^{13}C NMR (*trans*, 101 MHz, 330 K, $CDCl_3$): δ 157.6, 140.5, 128.7, 128.2, 126.4, 52.1, 32.6, 25.3, 16.0; HRMS: *m/z* [MH^+] calcd for $C_{11}H_{14}NO_2$: 192.10245; found: 192.10224.

Methyl *cis*-2-mesitylcyclopropylcarbamate 12

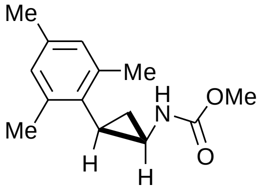

83% Yield, (*cis*:*trans*, 11:1); White crystals, mp 81.3-81.5 °C (hexane-EtOAc); IR (neat): ν_{max} 3349, 1686, 1531 cm^{-1}; ^{1}H NMR (*cis*, 400 MHz, 330 K, $CDCl_3$): δ 6.86 (s, 2 H), 4.11 (br, 1 H), 3.62 (s, 3 H), 3.17 (br m, 1 H), 2.34 (s, 6 H), 2.26 (s, 3 H), 1.98 (br q, *J* = 8.3 Hz, 1 H), 1.51 (m, 1 H), 0.79 (ddd, *J* = 7.8, 6.0, 3.9 Hz, 1 H); ^{13}C NMR (*cis*, 126 MHz, $CDCl_3$): δ 157.4, 138.6, 136.3, 129.5, 51.9, 28.0, 20.7, 20.4, 17.5, 16.3; HRMS: *m/z* [MH$^+$] calcd for $C_{14}H_{19}NO_2$: 233.14103; found: 233.14112.

Methyl *cis*-2-(4-fluorophenyl)cyclopropylcarbamate 13

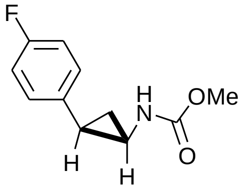

85% Yield (*cis*:*trans*, 9:1); White crystals, mp 91.8-92.2 °C (hexane-EtOAc); IR (neat): ν_{max} 3348, 1690, 1526, 1508 cm^{-1}; ^{1}H NMR (*cis*, 400 MHz, 330 K, $CDCl_3$): δ 7.16 (dd, *J* = 8.7, 5.4 Hz, 2 H), 6.99 (t, *J* = 8.7, 2 H), 4.27 (br, 1 H), 3.59 (s, 3 H), 2.95 (tt, 7.4, 3.8 Hz, 1 H), 2.23 (br q, *J* = 7.9 Hz, 1 H), 1.32 (ddd, *J* = 9.1, 7.2, 6.4 Hz, 1 H), 0.98 (td, *J* = 6.6, 4.3 Hz, 1 H); ^{13}C NMR (*cis*, 126 MHz, $CDCl_3$): δ 161.83 (d, *J* = 245.0 Hz), 157.5, 132.0 (d, *J* = 2.9 Hz), 130.3 (d, *J* = 7.9 Hz), 115.1 (d, *J* = 21.4 Hz), 51.9, 29.2, 21.5, 12.1; ^{1}H NMR (*trans*, 400 MHz, 330 K, $CDCl_3$): δ 7.16 (dd, *J* = 8.7, 5.4 Hz, 2 H), 6.99 (t, *J* = 8.7, 2 H), 4.87 (s, 1 H), 3.71 (s, 3 H), 2.73-2.67 (m, 1 H), 2.07 (ddd, J = 9.8, 6.5, 3.4 Hz, 1 H), 1.19-1.10 (m, 1 H); HRMS: *m/z* [MH$^+$] calcd for $C_{11}H_{12}NO_2F$: 209.08466; found: 209.08562.

Methyl *cis*-2-(4-chlorophenyl)cyclopropylcarbamate 14

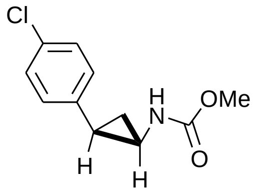

79% Yield, (*cis*:*trans*, 10:1); White solid, mp 61-64 °C; IR (neat): ν_{max} 3257, 1708 cm^{-1}; ^{1}H NMR (*cis*, 400 MHz, 330 K, $CDCl_3$): δ 7.27 (d, *J* = 8.2 Hz, 2 H), 7.13 (d, *J* = 8.3 Hz, 2 H), 4.30 (br, 1 H), 3.59 (s, 3 H), 3.01-2.93 (m, 1 H), 2.22 (br q, *J* = 7.9 Hz, 1 H), 1.33 (ddd, *J* = 9.1, 7.2, 6.4 Hz, 1 H), 1.00 (td, *J* = 6.6, 4.4 Hz, 1 H); ^{13}C NMR (126 MHz, $CDCl_3$): δ 157.4, 134.8, 132.2, 130.0, 128.3, 52.0, 29.3, 21.5, 12.0; ^{1}H NMR (*trans*, 400 MHz, 330 K, $CDCl_3$): δ 7.27 (d, *J* = 8.2 Hz, 2 H), 7.13 (d, *J* = 8.3 Hz, 2 H), 4.88 (br, 1 H), 3.71 (s, 3 H), 2.74-2.69 (m, 1 H), 2.10-2.03 (m, 1 H), 1.19-1.14 (m, 2 H); HRMS: *m/z* [MH^+] calcd for $C_{11}H_{12}NO_2Cl$: 225.05511; found: 225.05578.

Methyl *cis*-2-(4-(trifluoromethyl)phenyl)cyclopropylcarbamate 15

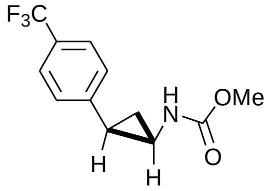

77% Yield, (*trans*:*cis*, 6:1); White crystals, mp 95.2-95.4 °C (hexane-EtOAc); IR (neat): ν_{max} 3348, 1692 cm^{-1}; ^{1}H NMR (*cis*, 400 MHz, 330 K, $CDCl_3$): δ 7.55 (d, *J* = 8.1 Hz, 2 H), 7.31 (d, *J* = 8.5 Hz, 2 H), 4.34 (br, 1 H), 3.58 (s, 3 H), 3.03 (tdd, 7.4, 4.3, 3.2 Hz, 1 H), 2.30 (br q, *J* = 7.8 Hz, 1 H), 1.40 (ddd, *J* = 9.0, 7.3, 6.5 Hz, 1 H), 1.10 (td, *J* = 6.7, 4.5 Hz, 1 H) ; ^{13}C NMR (*cis*, 101 MHz, 330 K, $CDCl_3$): δ 157.4, 140.9, 129.0, 126.9, 125.1 (q, J = 3.8 Hz), 124.3 (q, J = 271.7 Hz), 52.0, 30.0, 22.4, 12.5; ^{1}H NMR (*trans*, 400 MHz, 330 K, $CDCl_3$): δ 7.55 (d, *J* = 8.1 Hz, 2 H), 7.31 (d, *J* = 8.5 Hz, 2 H), 4.90 (br, 1 H), 3.71 (s, 3 H), 2.81-2.76 (m, 1 H), 2.17-2.12 (m, 1 H), 1.27-1.22 (m, 2H); HRMS: *m/z* [MH^+] calcd for $C_{12}H_{13}NO_2F_3$: 260.08983; found: 260.08983.

Methyl *cis*-2-(3, 4-difluorophenyl)cyclopropylcarbamate 16

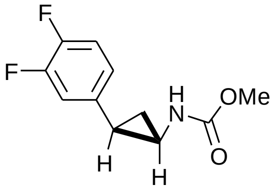

78% Yield, (*cis*:*trans*, 11:1); After recrystallisation, the *cis*-amidocyclopropane was isolated as a single isomer (264 mg, 1.16 mmol, 58%); White crystals, mp 106.6-107.2 °C (hexane-EtOAc); IR (neat): ν_{max} 3342, 1685, 1532, 1516 cm^{-1}; ^{1}H NMR (*cis*, 400 MHz, 330 K, $CDCl_3$): δ 7.09-6.93 (m, 2 H), 6.93-6.87 (m, 1 H), 4.50 (br, 1 H), 3.56 (s, 3 H), 2.93 (tt, *J* = 6.9, 3.6 Hz, 1 H), 2.17 (br q, *J* = 7.8 Hz, 1 H), 1.31 (ddd, *J* = 9.0, 7.4, 6.5 Hz, 1 H), 0.95 (td, *J* = 6.7, 4.4 Hz, 1 H); ^{13}C NMR (*cis*, 101 MHz, 330 K, $CDCl_3$): δ 157.5, 150.1 (dd, *J* = 246.3, 12.5 Hz), 149.2 (dd, *J* = 245.4, 12.7 Hz), 133.7 (dd, *J* = 5.9, 3.8 Hz), 124.7 (dd, *J* = 5.8, 3.3 Hz), 117.6 (d, *J* = 17.5), 116.8 (d, *J* = 17.2 Hz), 52.0, 29.5, 21.8, 12.2; HRMS: *m/z* [MH^+] calcd for $C_{11}H_{12}NO_2F_2$: 228.08361; found: 228.08361.

Methyl *cis*-2-(2-bromophenyl)cyclopropylcarbamate 17

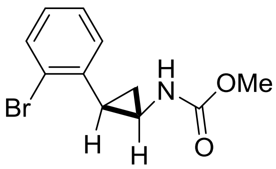

54% Yield, (*cis*:*trans*, 14:1); pale yellow oil; IR (neat) ν_{max} 3321, 3058, 3012, 2952, 1702, 1503; ^{1}H NMR (*cis*, 400 MHz, 330 K, $CDCl_3$): δ 7.58 (d, *J* = 8.0 Hz, 1 H), 7.25 (t, *J* = 7.5, 1 H), 7.12 (m, 2 H), 4.40 (br, 1 H), 3.57 (s, 3 H), 3.10-3.02 (m, 1 H), 2.33 (br q, *J* = 7.7 Hz, 1 H), 1.33 - 1.43 (m, 1 H), 1.09 - 1.17 (m, 1 H); ^{13}C NMR (cis, 100 MHz, $CDCl_3$) δ 157.5, 136.0, 132.6, 130.2, 128.3, 127.3, 51.9, 29.1, 23.4, 12.8; HRMS: *m/z* For [M-H^+] calcd for $C_{14}H_{18}O_3N$: 248.1282; found: 248.1281

Methyl *endo*-1,1a,6,6a-Tetrahydro-cyclopropaindencarbamate 18

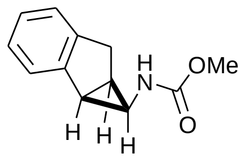

80% yield, (*endo*:*exo*, >20:1); Colorless oil; IR (neat): ν_{max} 3323, 1713, 1509 cm^{-1}; ^{1}H NMR (*endo*, 400 MHz, 330 K, $CDCl_3$): δ 7.30-7.25 (m, 2 H), 7.15-7.10 (m, 2 H), 3.90 (br, 1 H), 3.58 (s, 3 H), 3.21 (dd, *J* = 17.6, 7.1 Hz, 1 H), 3.10 (br, 1 H), 2.81 (d, *J* = 17.6 Hz, 1 H), 2.74 (ddd, *J* = 7.8, 6.5, 1.7 Hz, 1 H), 2.08 (q, *J* = 6.8 Hz, 1 H); ^{13}C NMR (126 MHz, $CDCl_3$): δ 157.5, 143.9, 139.0, 126.2, 126.1, 124.9, 123.8, 51.7, 32.1, 30.7, 28.6, 21.8; ; HRMS: *m/z* [MH^+] calcd for $C_{12}H_{13}NO_2$: 203.09408; found: 203.09379.

Methyl *endo*-bicyclo[4. 1.0]heptan-7-ylcarbamate 19

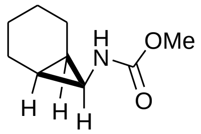

81% Yield (*endo*:*exo* 2.9:1); Pale yellow oil; IR (neat): ν_{max} 3320, 1701 cm^{-1}; ^{1}H NMR (*endo*, 400MHz, 330K, $CDCl_3$): δ 4.56 (br, 1 H), 3.68 (s, 3 H), 2.50 (td, *J* = 7.6, 2.6 Hz, 1 H), 1.93-1.80 (m, 2 H), 1.41-1.34 (m, 2 H), 1.30-0.96 (m, 6H); ^{13}C NMR (*endo*, 100 MHz, $CDCl_3$): δ 157.9, 52.0, 29.7, 21.9, 17.9, 11.6; ^{1}H NMR (*exo*, 400MHz, 330K, $CDCl_3$): δ 4.81 (br, 1 H), 3.63 (s, 3 H), 2.23 (td, *J* = 3.2, 2.5 Hz, 1 H), 1.93-1.80 (m, 2 H), 1.72-1.65 (m, 2 H), 1.30-0.96 (m, 6H); ^{13}C NMR (*exo*, 100 MHz, $CDCl_3$): δ 157.6, 51.7, 34.7, 22.3, 21.3, 19.3; HRMS: *m/z* [M^+] calcd for $C_9H_{15}NO_2$: 169.10973; found: 169.10888.

(±)-Methyl (2*R*,3*R*)-2,3-dibutylcyclopropylcarbamate 20

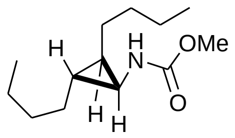

95% Yield (single isomer); Colorless oil; IR (neat): ν_{max} 3322, 1700 cm^{-1}; ^{1}H NMR (*trans*, 400MHz, 330K, $CDCl_3$): δ 4.57 (br, 1 H), 3.69 (s, 3 H), 2.37 (dt, *J* = 7.0, 3.4 Hz, 1 H), 1.50-1.31 (m, 10 H), 1.29-1.17 (m, 2 H), 0.93 (t, *J* = 7.1 Hz, 3 H), 0.92 (t, *J* = 7.1 Hz, 3 H), 0.66 (tt, *J* = 7.7, 5.8 Hz, 1 H), 0.47 (tdd, *J* = 6.4, 6.0, 3.7, 1 H); ^{13}C NMR (100 MHz, $CDCl_3$): δ 157.9, 51.9, 34.2, 32.2, 31.8, 31.0, 27.1, 26.6, 24.8, 22.5, 22.4, 13.9, 13.8; HRMS: *m/z* [M^+] calcd for $C_{13}H_{25}NO_2$: 227.18798; found: 227.18798.

(±)-Methyl (2*R*,3*S*)-2,3-diethylcyclopropylcarbamate 21

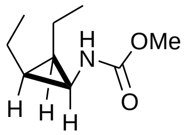

50% Yield (*cis*:*trans* 10:1); Colorless oil; IR (neat): ν_{max} 3328, 1708 cm^{-1}; ^{1}H NMR (*trans*, 400MHz, 330K, $CDCl_3$): δ 4.51 (br, 1 H), 3.62, (s, 3 H), 2.65 (td, *J* = 7.5, 3.3, 1 H), 1.35-1.27 (m, 4 H), 0.99 (t, *J* = 7.4 Hz, 3 H), 0.85-0.79 (m, 2 H); ^{13}C NMR (100 MHz, $CDCl_3$): δ 158.2, 51.8, 29.3, 20.7, 15.3, 13.8; HRMS: *m/z* [MH^+] calcd for $C_9H_{18}NO_2$: 172.13375; found: 172.13296.

Methyl *trans*-2-(trimethylsilyl)cyclopropylcarbamate 22

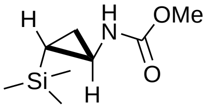

Yield 40%, (*trans*:*cis* >20:1); Colorless oil; IR (neat): ν_{max} 3321, 1702, 1247, 832; ^{1}H NMR (*trans*, 400 MHz, 330 K, $CDCl_3$): δ 4.76 (br, 1 H), 3.68 (s, 3H), 2.50 (ddd, *J* = 8.2, 6.2, 3.2 Hz, 1 H), 0.73 (ddd, *J* = 11.3, 4.6, 3.3 Hz, 1H), 0.63 (ddd, *J* = 8.1, 6.4, 4.6 Hz, 1H), 0.00 (s, 9 H), -0.17 (ddd, *J* = 11.3, 8.1, 4.9 Hz, 1 H); ^{13}C NMR (126 MHz, $CDCl_3$): 157.7, 51.93, 27.0, 10.5, 7.0, -2.6; HRMS: *m/z* [MH^+] calcd for $C_8H_{18}NO_2Si$: 188.11068; found: 188.11121.

Methyl *trans*-2-benzylcyclopropylcarbamate 23

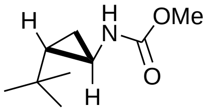

Yield 53%, (*trans*:*cis* >20:1); White crystals, mp 53.2-53.9 °C (hexane-EtOAc); IR (neat): ν_{max} 3304, 1697; ^{1}H NMR (*trans*, 400 MHz, 330 K, $CDCl_3$): δ 4.66 (br, 1 H), 3.69 (s, 3 H), 2.49 (dtd, J= 6.3, 3.7, 2.6 Hz, 1 H), 0.90 (s, 9 H), 0.83 (ddd, J = 9.6, 6.6, 3.7 Hz, 1 H), 0.70 (ddd, J = 7.2, 6.8, 5.7 Hz, 1H), 0.54 (ddd, J = 9.6, 5.7, 3.7 Hz, 1 H); ^{13}C NMR (126 MHz, $CDCl_3$): δ 157.6, 51.8, 31.9, 29.1, 28.2, 26.1, 10.0; HRMS: *m/z* [MH^+] calcd for C_9H_{18} NO_2: 172.13375; found: 172.13412.

cis-2-Phenylcyclopropanamine hydrogen iodide salt 24

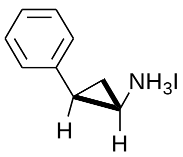

96% Yield; Brown solid, mp 175.6-175.8 °C; IR (neat): ν_{max} 3200-2750 (br); ^{1}H NMR (*cis*, 400 MHz, D_2O): δ 7.37-7.24 (m, 5 H), 4.70 (s, 3 H), 2.87 (td, *J* = 7.7, 4.3 Hz, 1 H), 2.48 (br q, *J* = 7.8 Hz, 1 H), 1.31 (dt, *J* = 9.4, 7.4 Hz, 1 H), 1.22 (td, *J* = 7.3, 4.3 Hz, 1 H); ^{13}C NMR (*cis*, 101 MHz, D_2O): δ 133.4, 129.8, 129.0, 127.9, 27.4, 19.5, 7.7; HRMS: *m/z* $[M\text{-}I]^+$ calcd for $C_9H_{11}N$: 133.08860; found: 133.08905.

cis-2-(4-chlorophenyl)cyclopropanamine hydrogen iodide salt 25

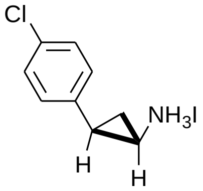

94% Yield; Yellow solid, mp 191.8-192.0 °C; IR (neat): ν_{max} 3200-2650 (br), 1575, 1491, 1470, 1438 cm^{-1}; ^{1}H NMR (*cis*, 400 MHz, D_2O): δ 7.33 (d, *J* = 8.5 Hz, 2 H), 7.26 (d, *J* = 8.5 Hz, 2 H), 4.70 (s, 3 H) 2.89 (td, *J* = 7.7, 4.3 Hz, 1 H), 2.44 (br q, *J* = 7.9 Hz, 1 H), 1.34 (dt, *J* = 9.3, 7.4 Hz, 1 H), 1.21 (td, *J* = 7.3, 4.3 Hz, 1 H); ^{13}C NMR (*cis*, 101 MHz, D_2O): δ 133.0, 132.1, 131.4, 128.9, 27.5, 19.1, 8.1; HRMS: *m/z* $[MH^+]$ calcd for $C_9H_{11}NCl$: 168.05800; found: 168.05800.

cis-2-(3, 4-difluorophenyl)cyclopropanamine hydrogen iodide salt 26

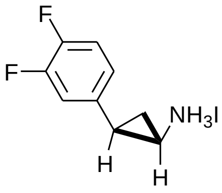

80% Yield; Yellow solid, mp 189.6-190.0 °C; IR (neat): ν_{max} 3060-2700 (br), 1564, 1518, 1490, 1425, 1274 cm^{-1}; ^{1}H NMR (*cis*, 400 MHz, D_2O): δ 7.26-7.11 (m, 2 H), 7.11-7.02 (m, 1 H), 4.70 (s, 3 H), 2.87 (td, *J* = 7.7, 4.3 Hz, 1 H), 2.45 (br q, *J* = 8.0 Hz, 1 H), 1.33 (dt, *J* = 9.4, 7.5 Hz, 1 H), 1.17 (td, *J* = 7.3, 4.3 Hz, 1 H); ^{13}C NMR (*cis*, 101 MHz, D_2O): δ 149.8 (dd, *J* = 244.9, 12.9 Hz), 149.6 (dd, *J* = 244.1, 12.3 Hz), 130.6 (dd, *J* = 6.2, 3.6 Hz), 126.4 (dd, *J* = 6.6, 3.5 Hz),

118.9 (d, *J* = 17.5 Hz), 117. 7 (d, *J* = 17.4 Hz), 27.6, 19.0, 8.3; HRMS: *m/z* $[M-I]^+$ calcd for $C_9H_{10}NF_2$: 170.07813; found: 170.07792.

trans-2-tert-butylcyclopropanamine hydrogen iodide salt 27

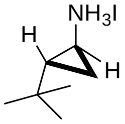

70% Yield; Pale yellow solid, mp 209.8-210.0 °C; IR (neat): ν_{max} 3150-2700 (br); ^{1}H NMR (*trans*, 400 MHz, D_2O): δ 4.69 (s, 3 H), 2.45 (dt, *J* = 7.8, 3.9 Hz, 1 H), 1.04 (ddd, *J* = 10.7, 7.1, 3.9 Hz, 1 H), 0.78-0.75 (m, 10H), 0.70 (ddd, *J* = 10.6, 6.9, 4.0 Hz, 1 H); ^{13}C NMR (*trans*, 101 MHz, D_2O): δ 28.5, 28.2, 27.2, 25.1, 6.3; HRMS: *m/z* $[M-I]^+$ calcd for $C_7H_{16}N$: 114.12827; found: 114.12881.

Benzyl *trans*-2-*tert*-butylcyclopropylcarbamate 28

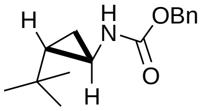

59% Yield (*trans*:*cis* >20:1); Colorless oil; IR (neat): ν_{max} 3319, 1696, 1521, 1498 cm^{-1}; ^{1}H NMR (*trans*, 400 MHz, $CDCl_3$): δ 7.39-7.28 (m, 5 H), 5.14-5.11 (m, 2 H), 4.97 (br, 1 H), 2.51 (dq, J = 6.9, 3.5 Hz, 1 H), 0.89 (s, 9 H), 0.83 (ddd, J = 9.9, 6.7, 3.7 Hz, 1 H), 0.69 (br q, *J* = 6.6 Hz, 1 H), 0.55 (ddd, *J* = 9.9, 5.6, 3.7 Hz, 1 H); ^{13}C NMR (*trans*, 101 MHz, $CDCl_3$): δ 156.9, 136.8, 128.4, 128.0, 127.9, 66.5, 32.2, 29.0, 28.3, 26.4, 10.0; HRMS: *m/z* $[M-I]^+$ calcd for $C_{15}H_{22}NO_2N$: 248.16505; found: 248.16447.

References

[1] S. J. Cho, N. H. Jensen, t. Kurome, S. Kadari, M. L. Manzano, J. E. Malberg, B. Caldarone, B. L. Roth, A. P. Kozikowski, *J. Med.. Chem..* **2009**, *52*, 1885.

[2] a) G. Bégis, D. E. Cladingboel, W. B. Motherwell, *Chem. Commun.* **2003**, 2656; b) W. B. Motherwell, G. Bégis, D. E. Cladingboel, L. Jerome, T. D. Sheppard, *Tetrahedron* **2007**, *63*, 6462; c) G. Bégis, T. D. Sheppard, D. E. Cladingboel, W. B. Motherwell, D. A. Tocher, *Synthesis* **2005**, 3186; d) G. Bégis, D. E. Cladingboel, L. Jerome, W. B. Motherwell, T. D. Sheppard, *Eur. J. Org. Chem.* **2009**, 1532.

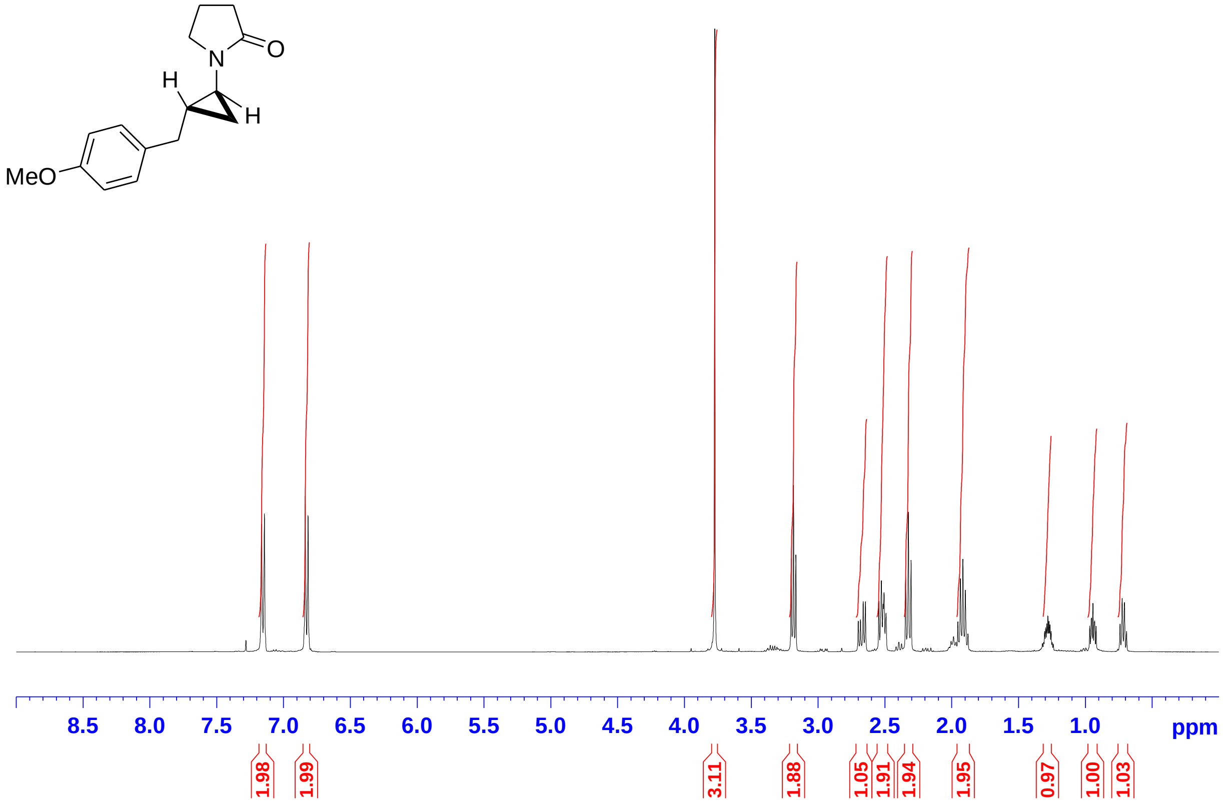
MeO
N
O
H
H
8.5 8.0 7.5 7.0 6.5 6.0 5.5 5.0 4.5 4.0 3.5 3.0 2.5 2.0 1.5 1.0 ppm
1.98
1.99
3.11
1.88
1.05
1.91
1.94
1.95
0.97
1.00
1.03

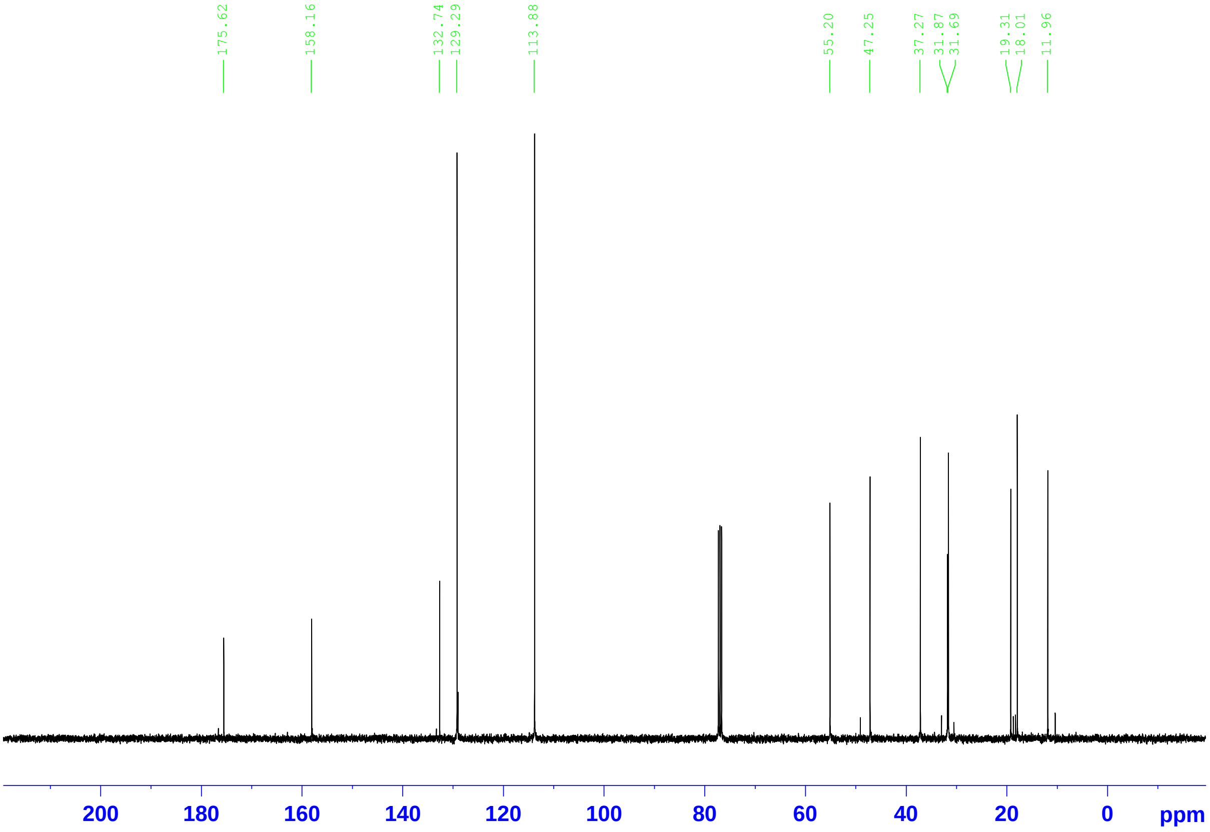
175.62
158.16
132.74
129.29
113.88
55.20
47.25
37.27
31.87
31.69
19.31
18.01
11.96
200 180 160 140 120 100 80 60 40 20 0 ppm

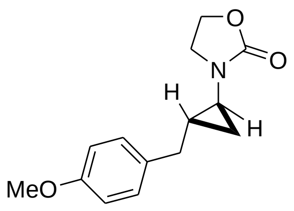
O
N
O
H
H
MeO

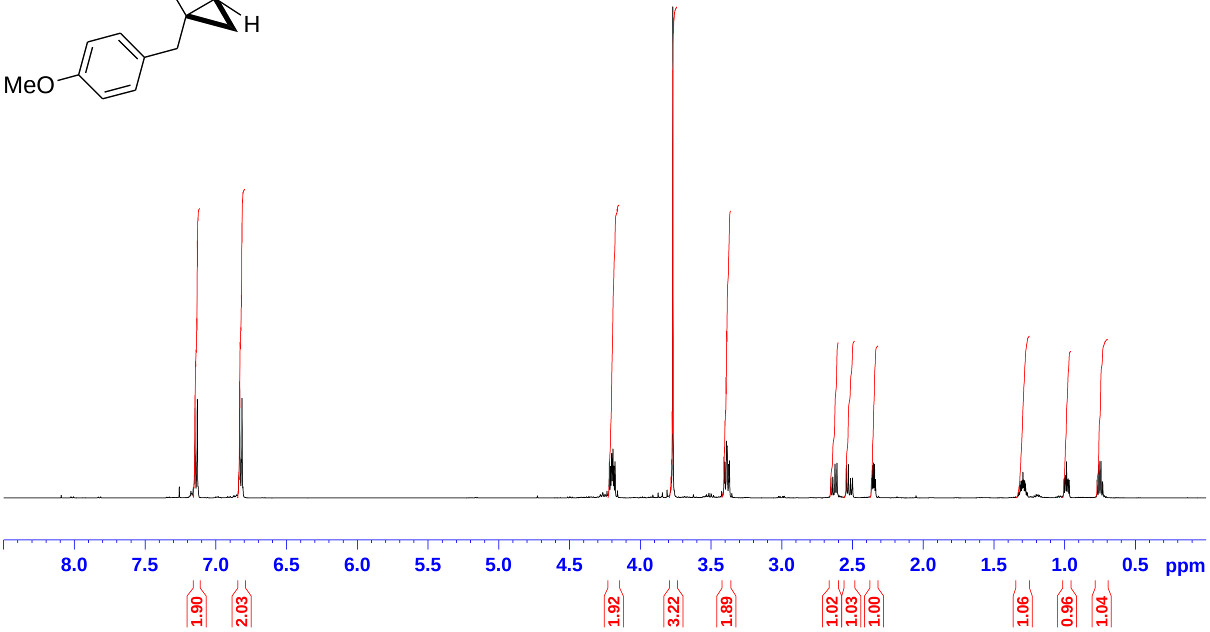
8.0 7.5 7.0 6.5 6.0 5.5 5.0 4.5 4.0 3.5 3.0 2.5 2.0 1.5 1.0 0.5 ppm
1.90 2.03 1.92 3.22 1.89 1.02 1.03 1.00 1.06 0.96 1.04

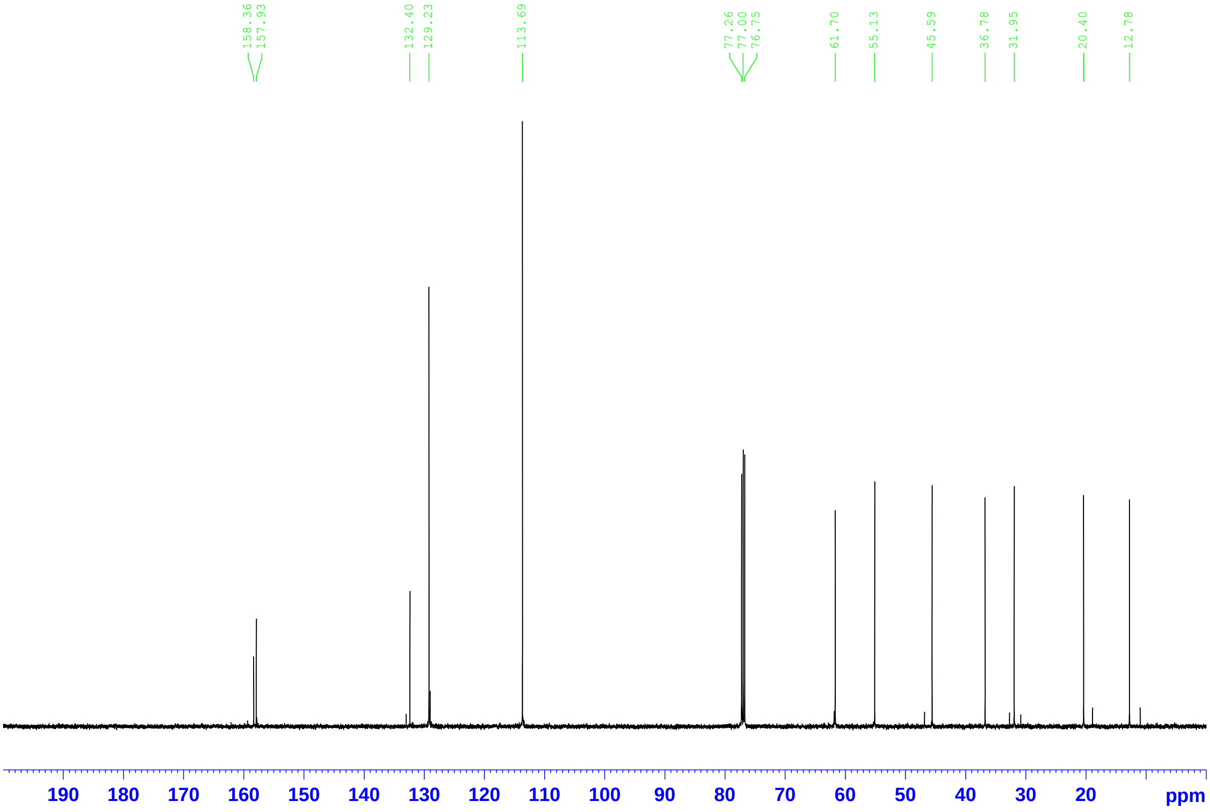
158.36 157.93 132.40 129.23 113.69 77.26 77.00 76.75 61.70 55.13 45.59 36.78 31.95 20.40 12.78
190 180 170 160 150 140 130 120 110 100 90 80 70 60 50 40 30 20 ppm

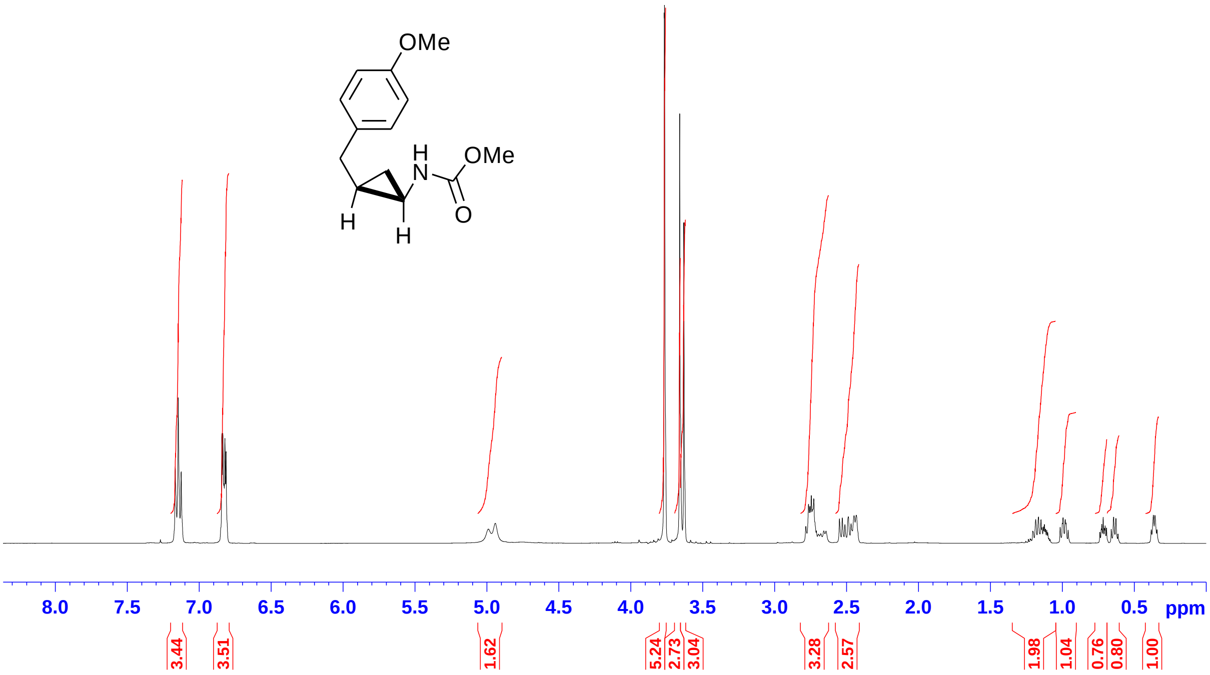
OMe
H
N
OMe
O
H
H
8.0 7.5 7.0 6.5 6.0 5.5 5.0 4.5 4.0 3.5 3.0 2.5 2.0 1.5 1.0 0.5 ppm
3.44 3.51 1.62 5.24 2.73 3.04 3.28 2.57 1.98 1.04 0.76 0.80 1.00

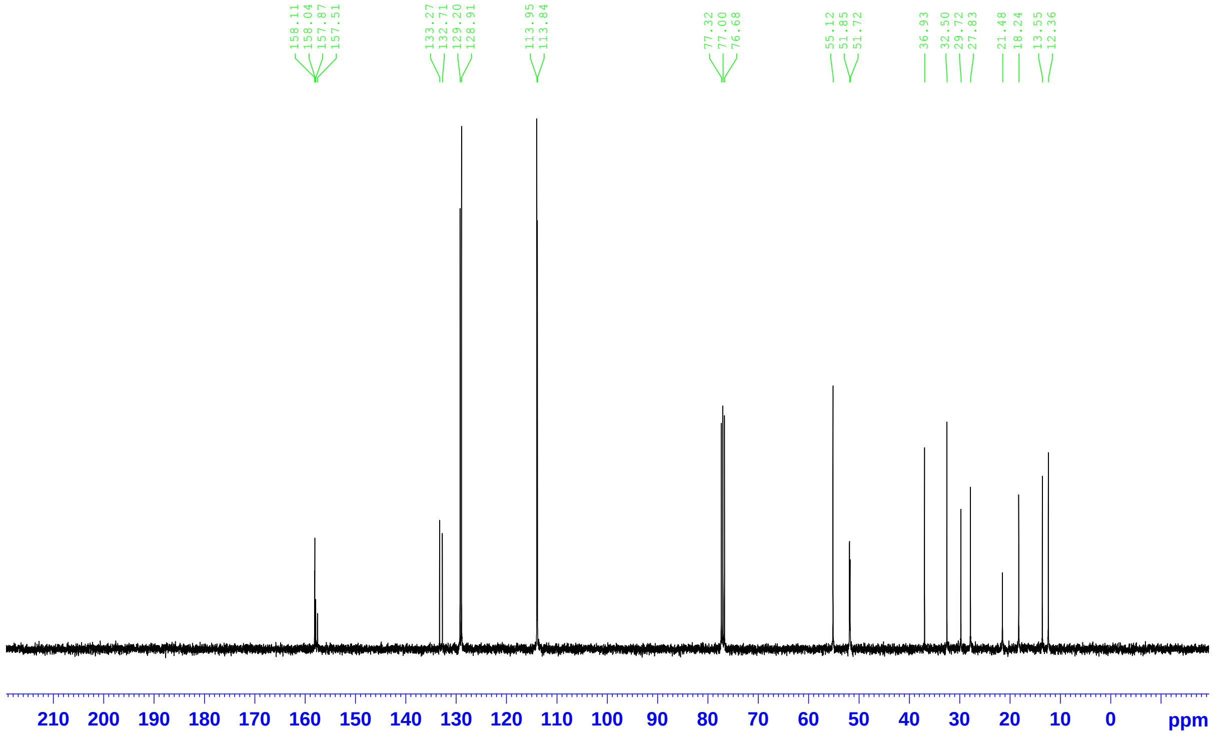
158.11 158.04 157.87 157.51
133.27 132.71 129.20 128.91
113.95 113.84
77.32 77.00 76.68
55.12 51.85 51.72
36.93 32.50 29.72 27.83
21.48 18.24 13.55 12.36
210 200 190 180 170 160 150 140 130 120 110 100 90 80 70 60 50 40 30 20 10 0 ppm

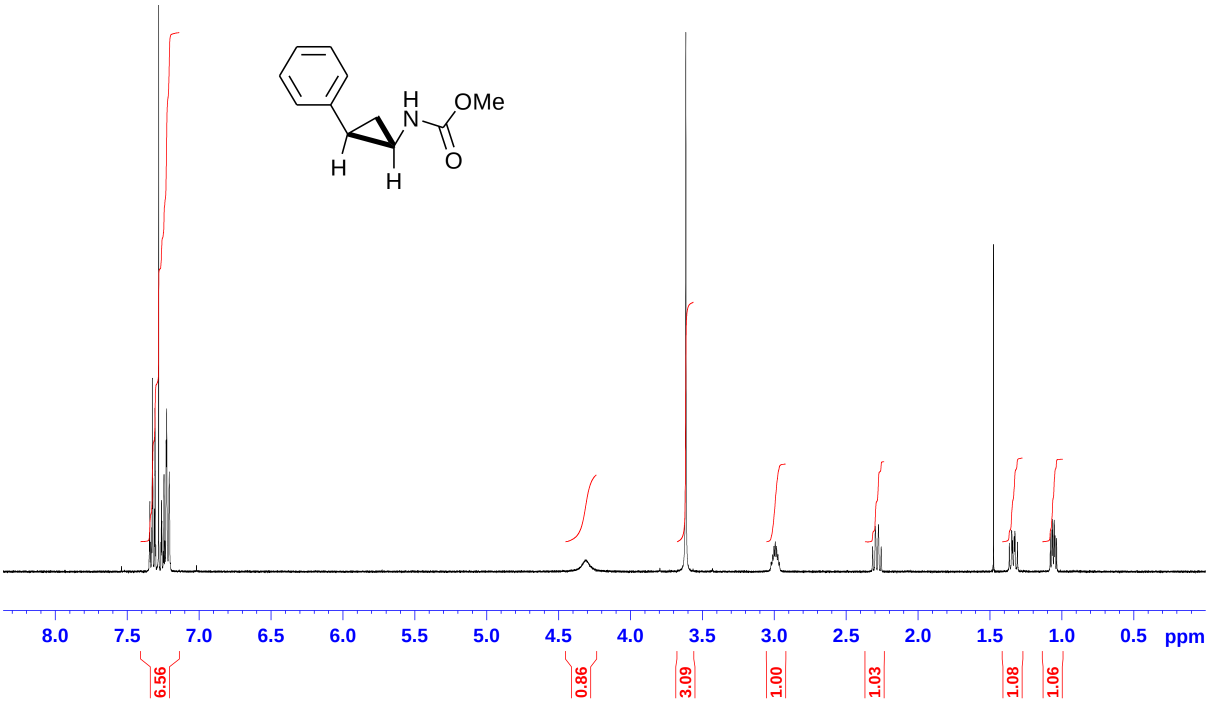
H
N
OMe
O
H
H
8.0 7.5 7.0 6.5 6.0 5.5 5.0 4.5 4.0 3.5 3.0 2.5 2.0 1.5 1.0 0.5 ppm
6.56
0.86
3.09
1.00
1.03
1.08
1.06

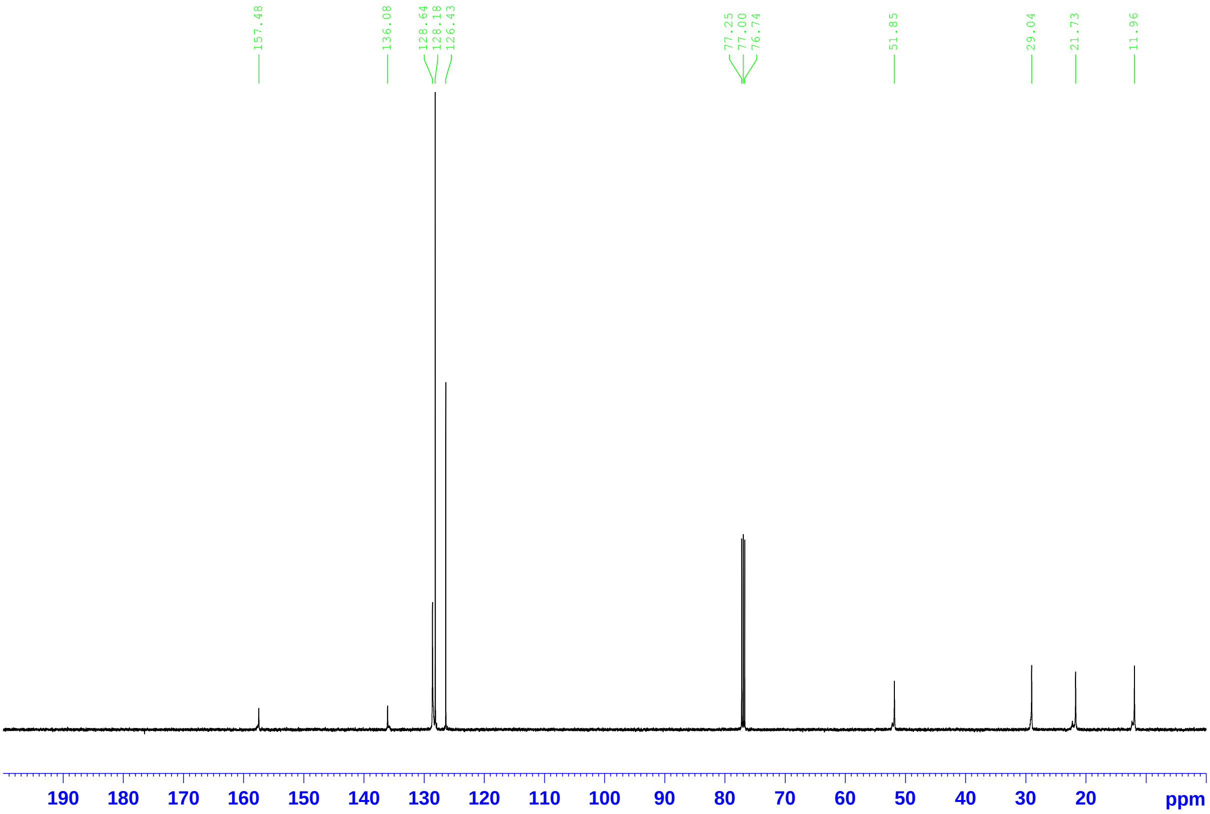
157.48
136.08
128.64
128.18
126.43
77.25
77.00
76.74
51.85
29.04
21.73
11.96
190 180 170 160 150 140 130 120 110 100 90 80 70 60 50 40 30 20 ppm

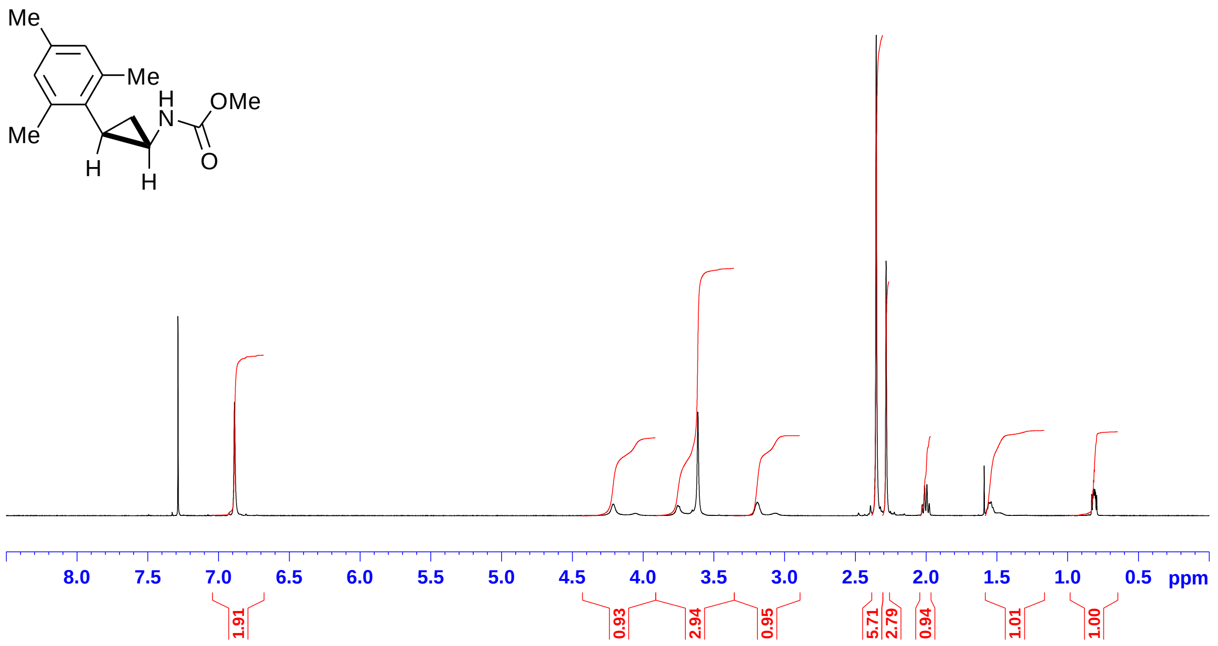
Me
Me
Me
H
N
OMe
O
H
H
8.0 7.5 7.0 6.5 6.0 5.5 5.0 4.5 4.0 3.5 3.0 2.5 2.0 1.5 1.0 0.5 ppm
1.91
0.93
2.94
0.95
5.71
2.79
0.94
1.01
1.00

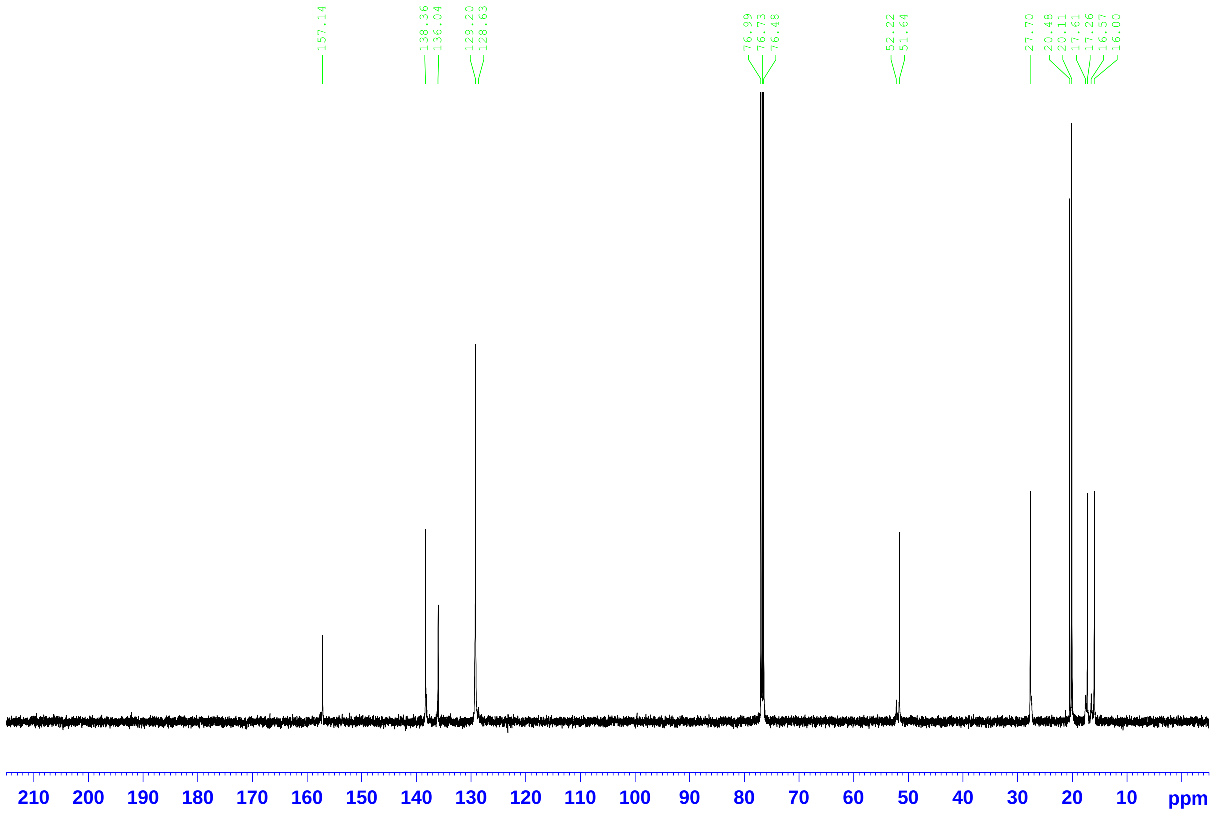
157.14
138.36
136.04
129.20
128.63
76.99
76.73
76.48
52.22
51.64
27.70
20.48
20.11
17.61
17.26
16.57
16.00
210 200 190 180 170 160 150 140 130 120 110 100 90 80 70 60 50 40 30 20 10 ppm

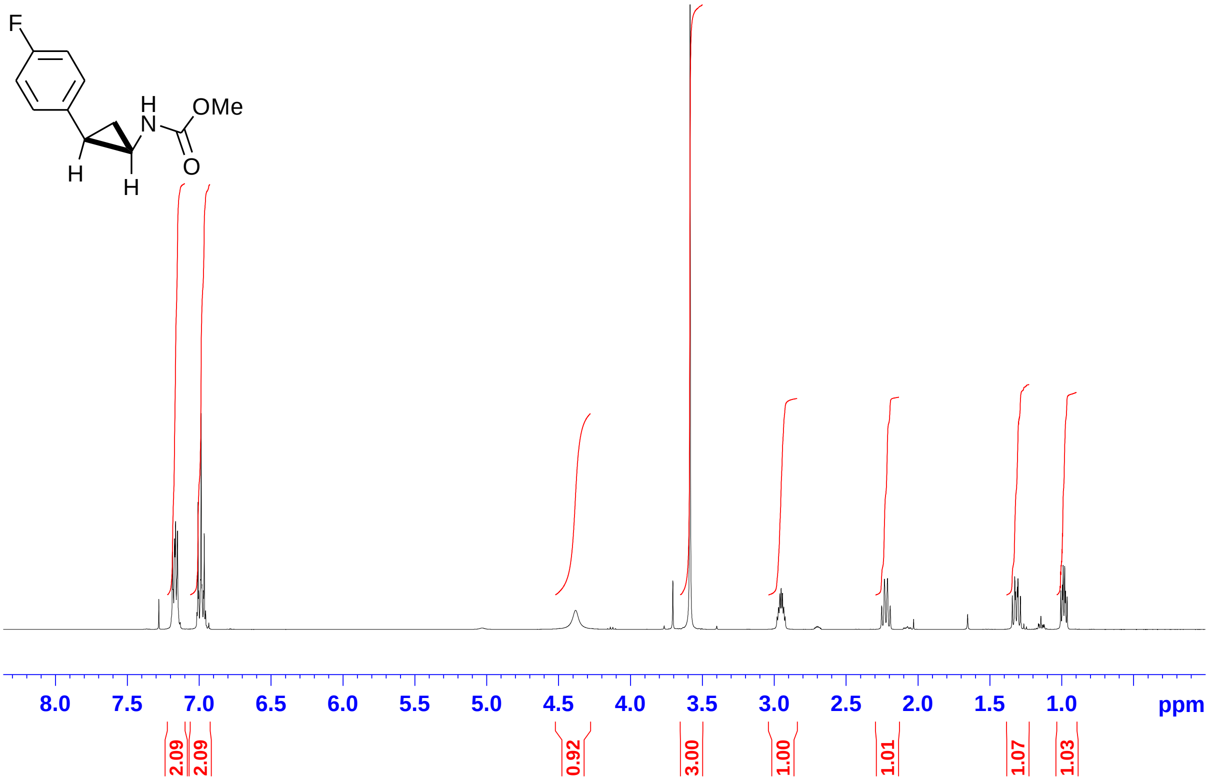
F
H
N
OMe
O
H
H
8.0 7.5 7.0 6.5 6.0 5.5 5.0 4.5 4.0 3.5 3.0 2.5 2.0 1.5 1.0 ppm
2.09 2.09 0.92 3.00 1.00 1.01 1.07 1.03

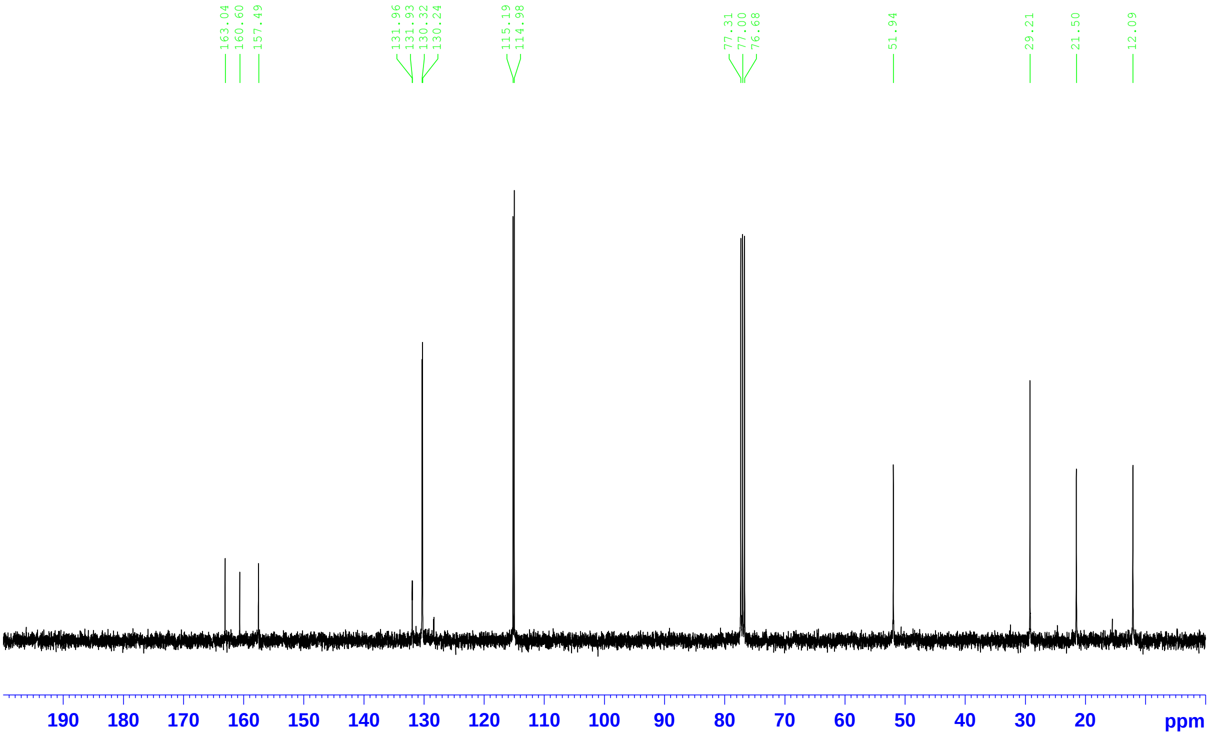
163.04
160.60
157.49
131.96
131.93
130.32
130.24
115.19
114.98
77.31
77.00
76.68
51.94
29.21
21.50
12.09
190 180 170 160 150 140 130 120 110 100 90 80 70 60 50 40 30 20 ppm

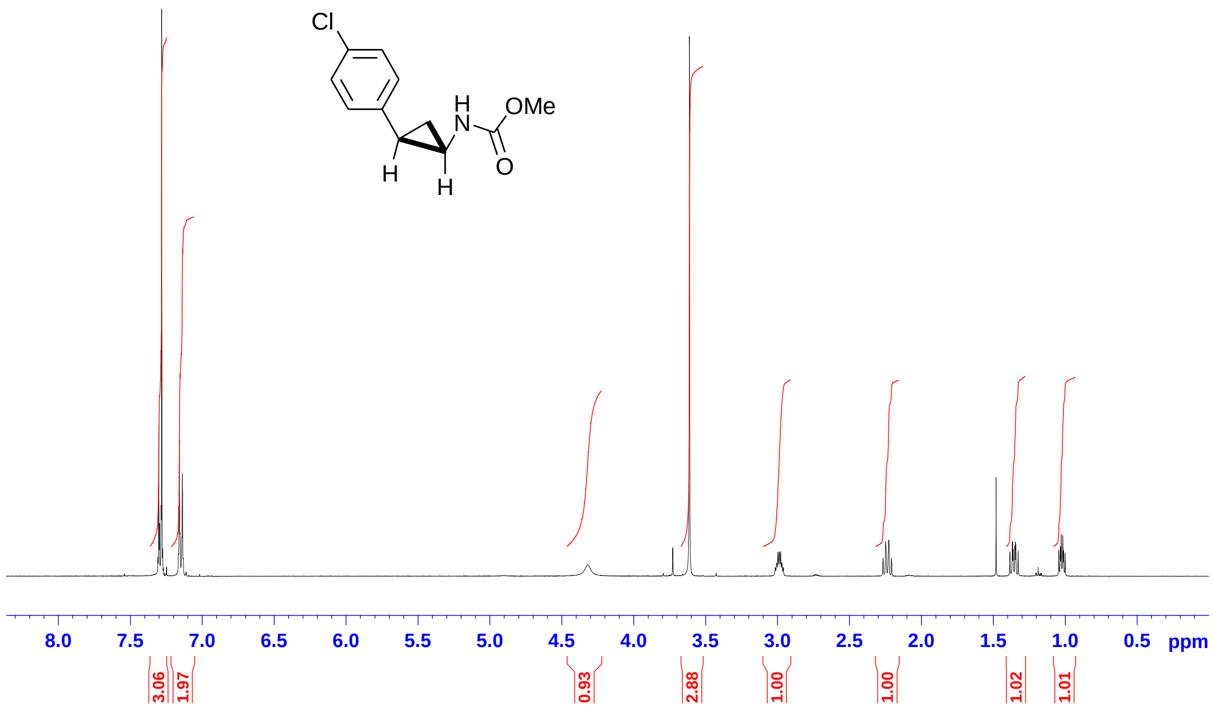
Cl
H
N
OMe
O
H
H
8.0 7.5 7.0 6.5 6.0 5.5 5.0 4.5 4.0 3.5 3.0 2.5 2.0 1.5 1.0 0.5 ppm
3.06 1.97 0.93 2.88 1.00 1.00 1.02 1.01

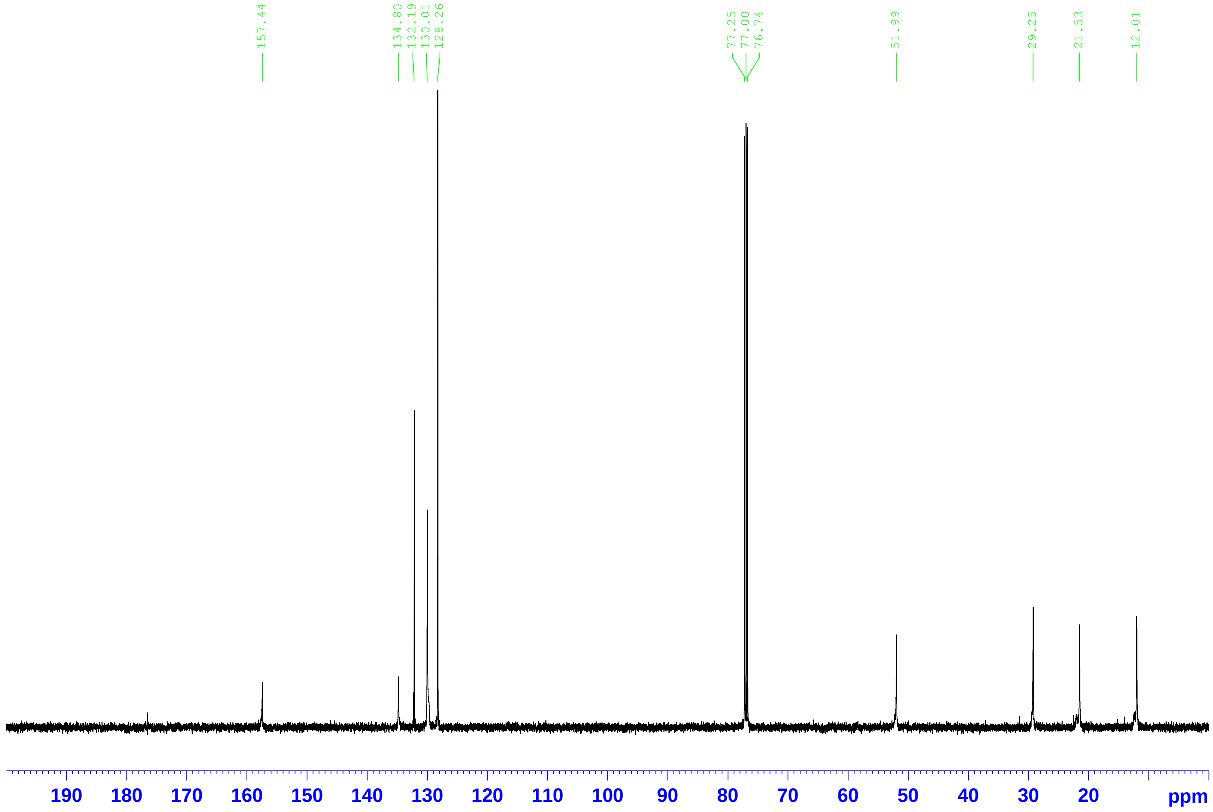
157.44
134.80
132.19
130.01
128.26
77.25
77.00
76.74
51.99
29.25
21.53
12.01
190 180 170 160 150 140 130 120 110 100 90 80 70 60 50 40 30 20 ppm

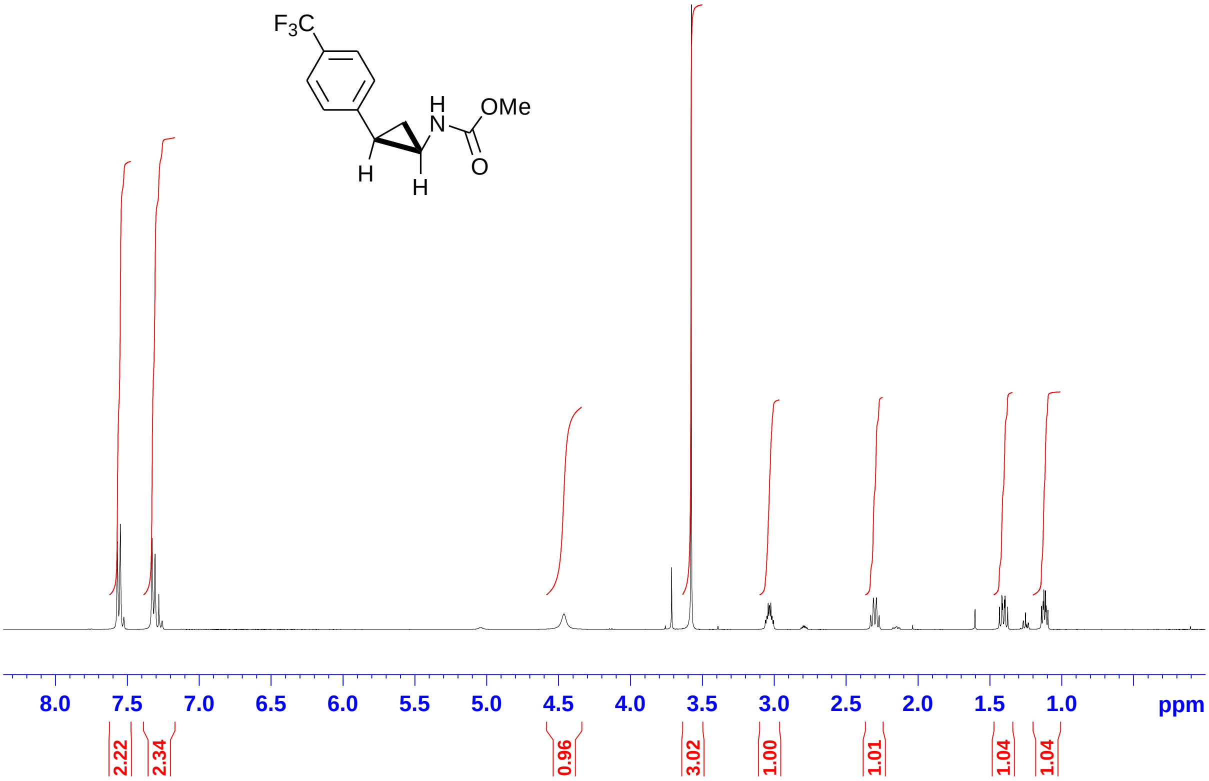
F3C
H
N
OMe
O
H
H
8.0
7.5
7.0
6.5
6.0
5.5
5.0
4.5
4.0
3.5
3.0
2.5
2.0
1.5
1.0
ppm
2.22
2.34
0.96
3.02
1.00
1.01
1.04
1.04

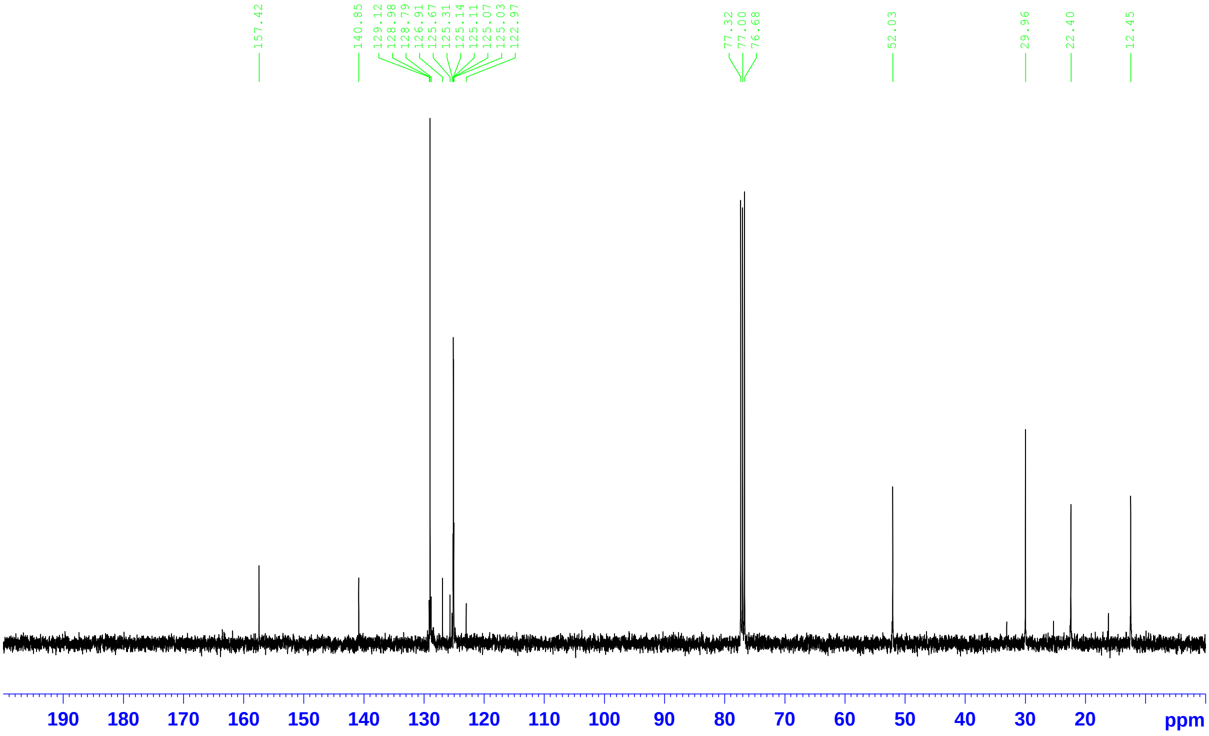
157.42
140.85
129.12
128.98
128.79
126.91
125.67
125.31
125.14
125.11
125.07
125.03
122.97
77.32
77.00
76.68
52.03
29.96
22.40
12.45
190
180
170
160
150
140
130
120
110
100
90
80
70
60
50
40
30
20
ppm

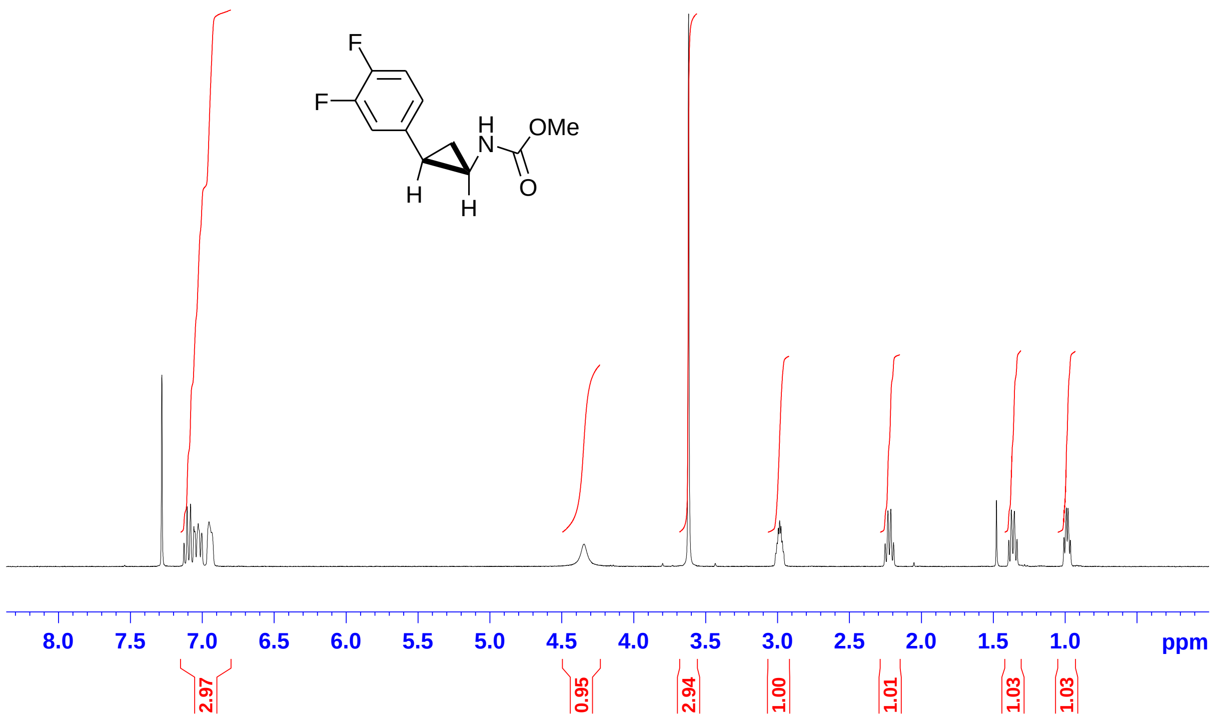
F
F
H
N
OMe
O
H
H
8.0
7.5
7.0
6.5
6.0
5.5
5.0
4.5
4.0
3.5
3.0
2.5
2.0
1.5
1.0
ppm
2.97
0.95
2.94
1.00
1.01
1.03
1.03

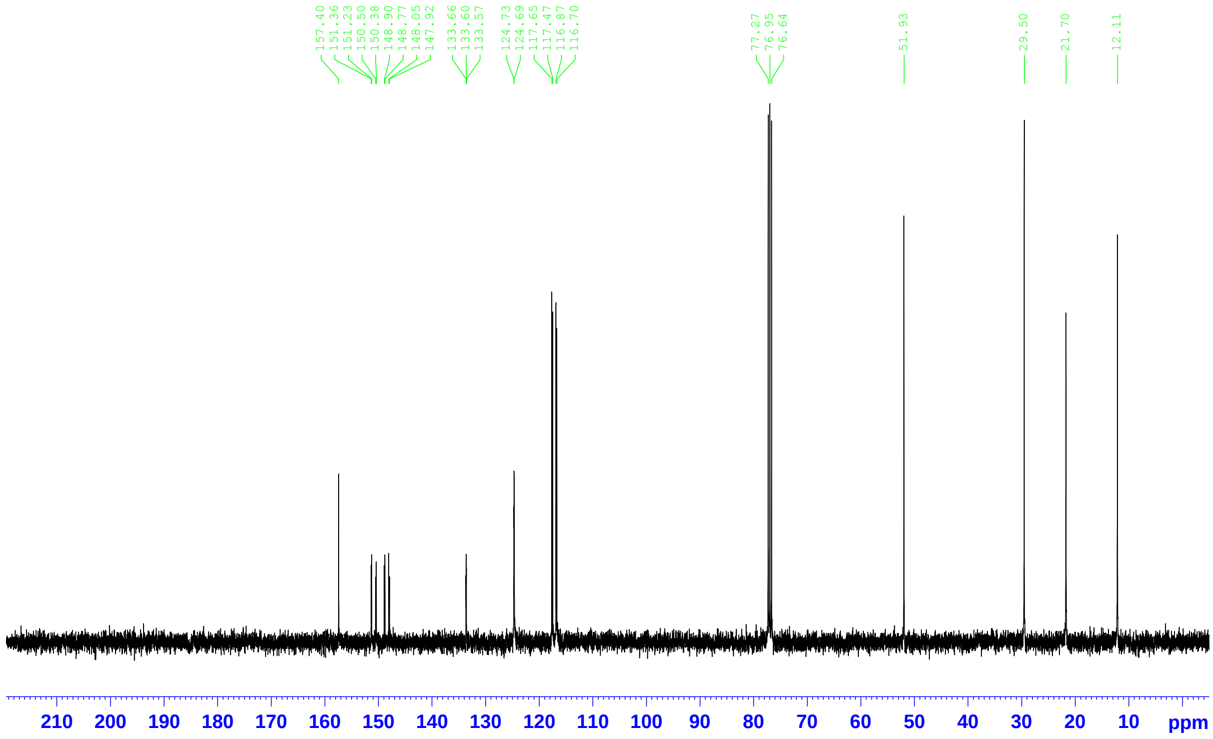
157.40
151.36
151.23
150.50
150.38
148.90
148.77
148.05
147.92
133.66
133.60
133.57
124.73
124.69
117.65
117.47
116.87
116.70
77.27
76.95
76.64
51.93
29.50
21.70
12.11
210
200
190
180
170
160
150
140
130
120
110
100
90
80
70
60
50
40
30
20
10
ppm

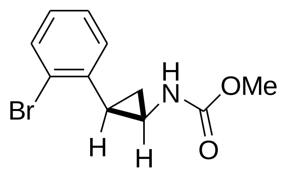
Br
H
N
OMe
H
H
O

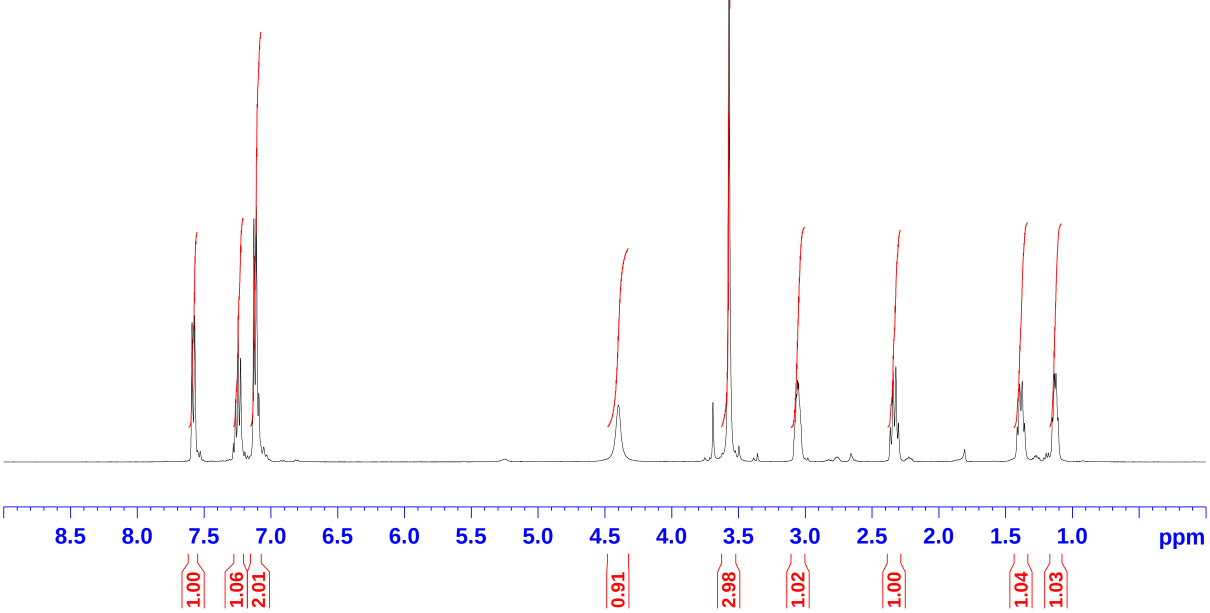
8.5
8.0
7.5
7.0
6.5
6.0
5.5
5.0
4.5
4.0
3.5
3.0
2.5
2.0
1.5
1.0
ppm
1.00
1.06
2.01
0.91
2.98
1.02
1.00
1.04
1.03

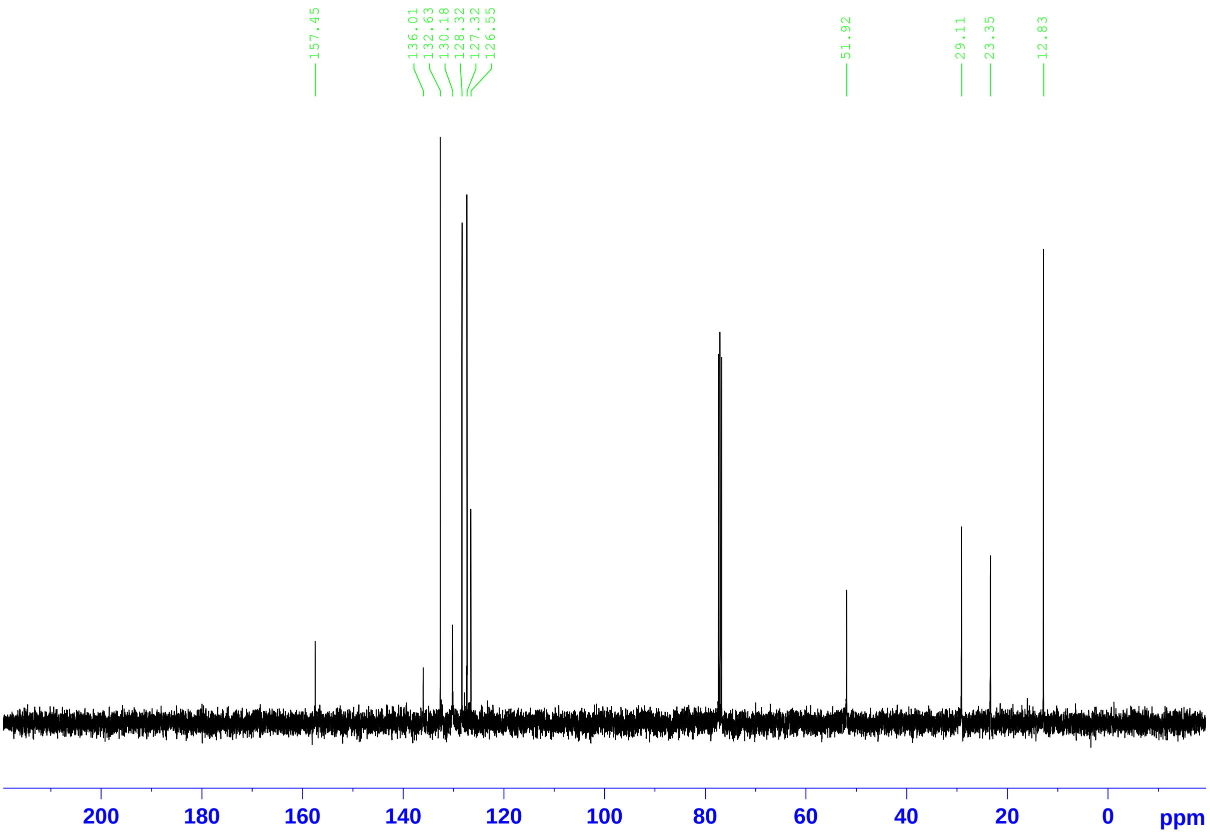
157.45
136.01
132.63
130.18
128.32
127.32
126.55
51.92
29.11
23.35
12.83
200
180
160
140
120
100
80
60
40
20
0
ppm

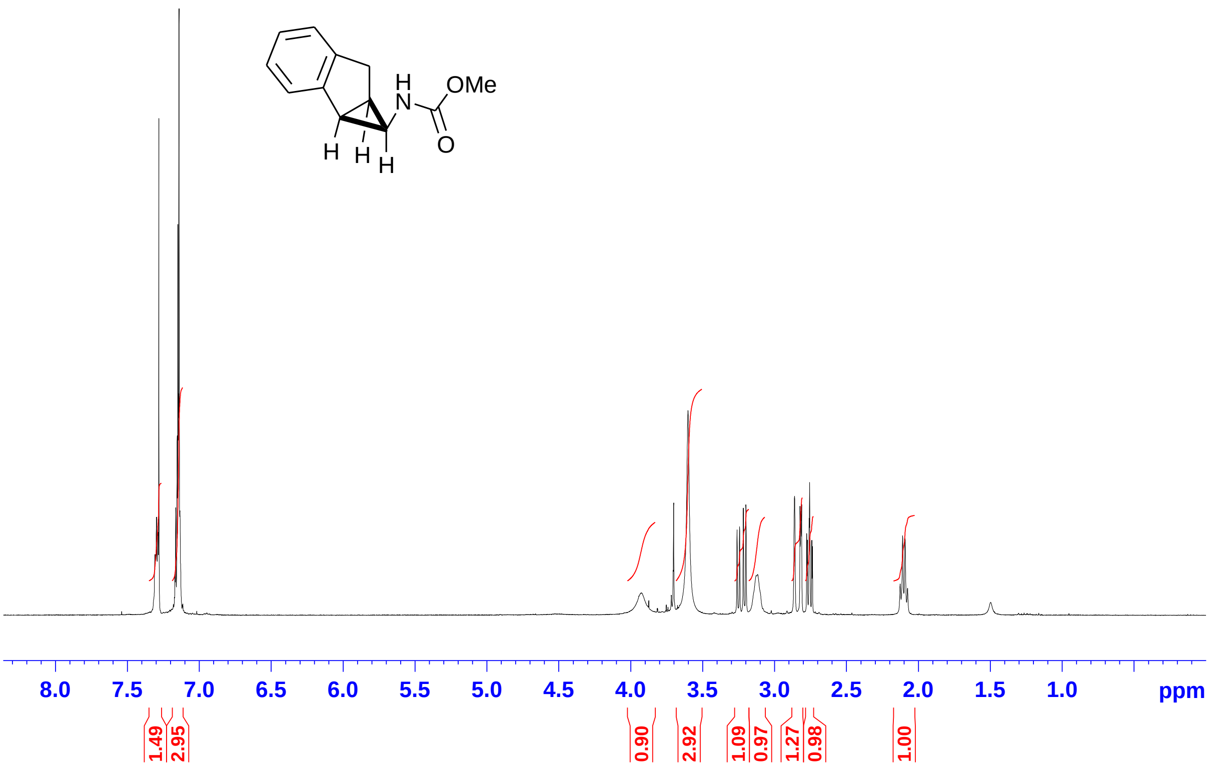
H
N
OMe
O
H H H
8.0 7.5 7.0 6.5 6.0 5.5 5.0 4.5 4.0 3.5 3.0 2.5 2.0 1.5 1.0 ppm
1.49 2.95 0.90 2.92 1.09 0.97 1.27 0.98 1.00

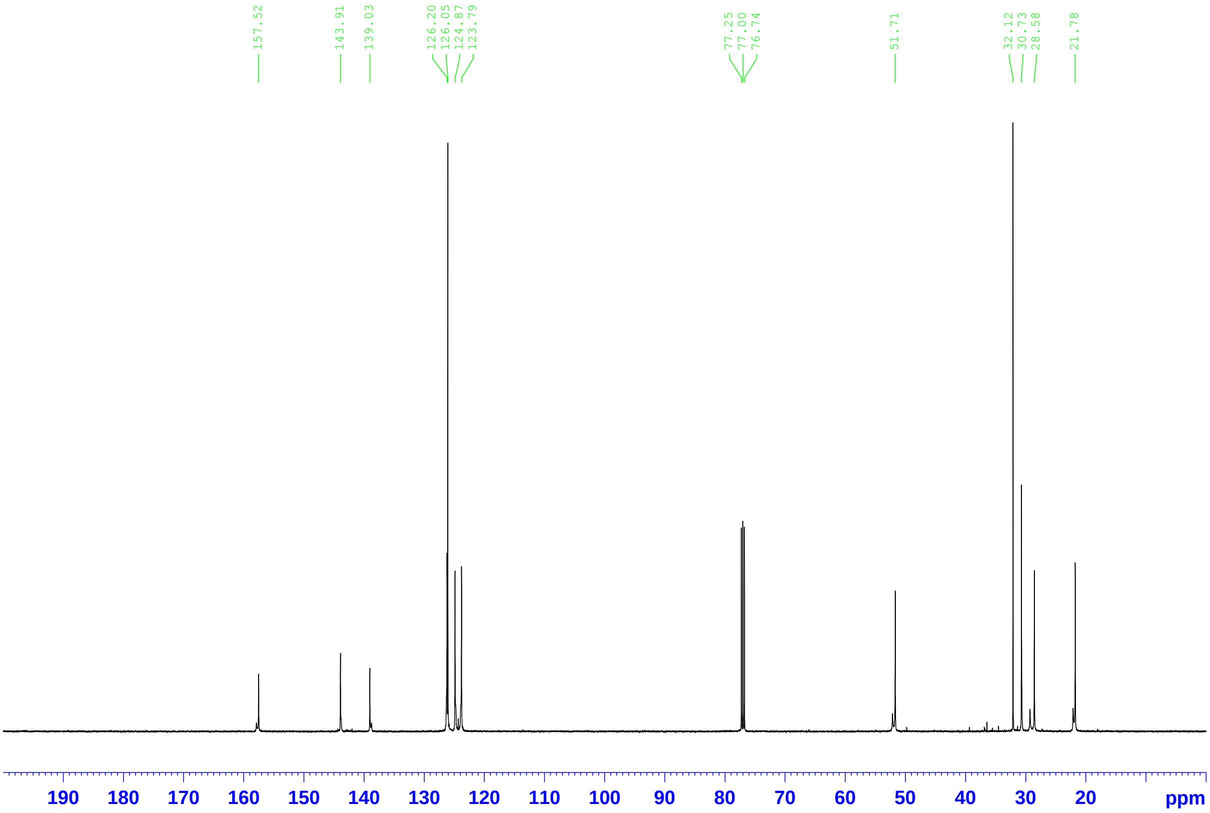
157.52
143.91 139.03
126.20 126.05 124.87 123.79
77.25 77.00 76.74
51.71
32.12 30.73 28.58
21.78
190 180 170 160 150 140 130 120 110 100 90 80 70 60 50 40 30 20 ppm

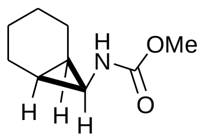
H
N
OMe
O
H
H
H

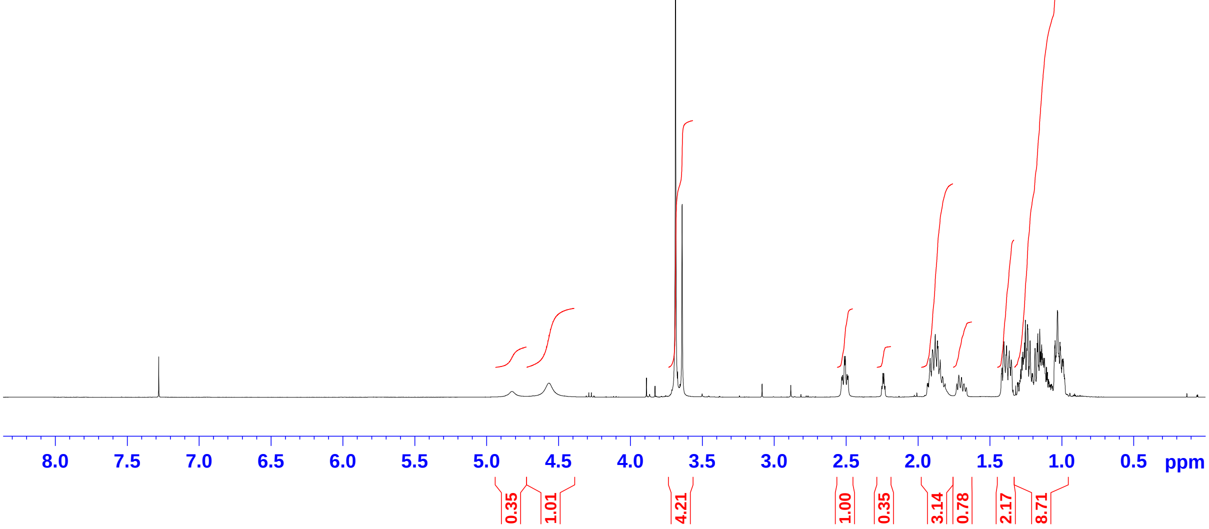
8.0 7.5 7.0 6.5 6.0 5.5 5.0 4.5 4.0 3.5 3.0 2.5 2.0 1.5 1.0 0.5 ppm
0.35
1.01
4.21
1.00
0.35
3.14
0.78
2.17
8.71

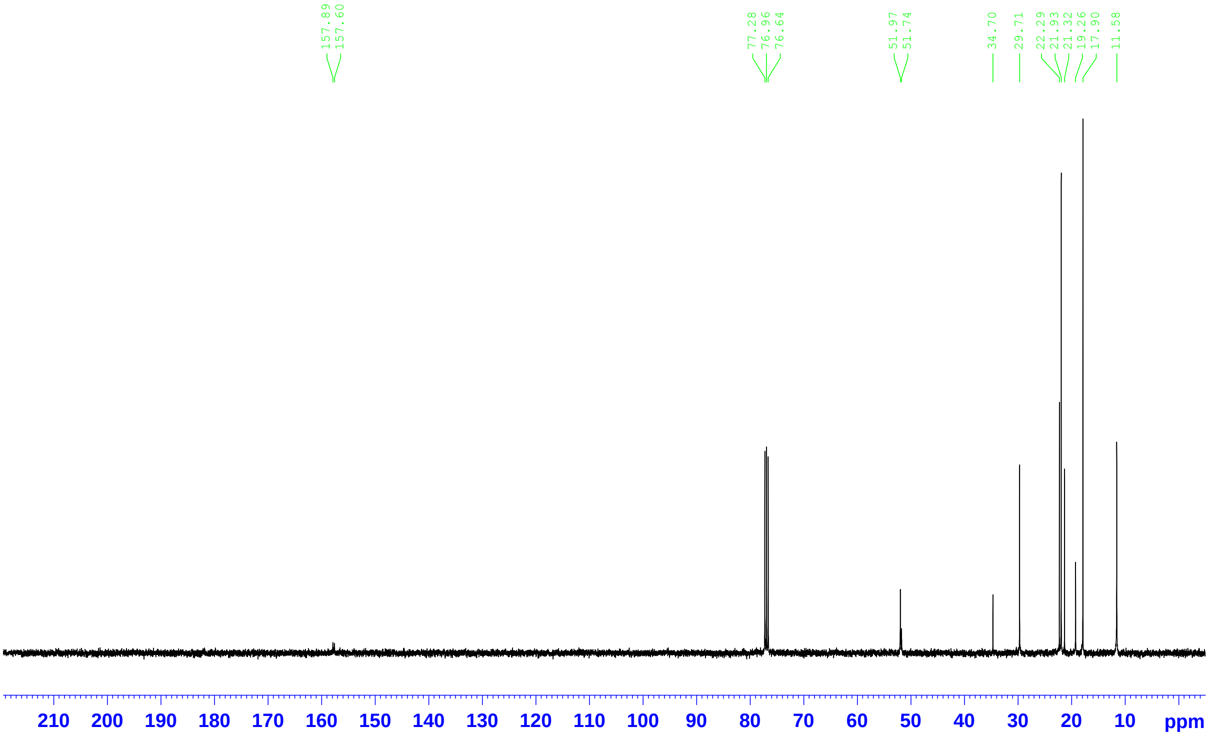
157.89
157.60
77.28
76.96
76.64
51.97
51.74
34.70
29.71
22.29
21.93
21.32
19.26
17.90
11.58
210 200 190 180 170 160 150 140 130 120 110 100 90 80 70 60 50 40 30 20 10 ppm

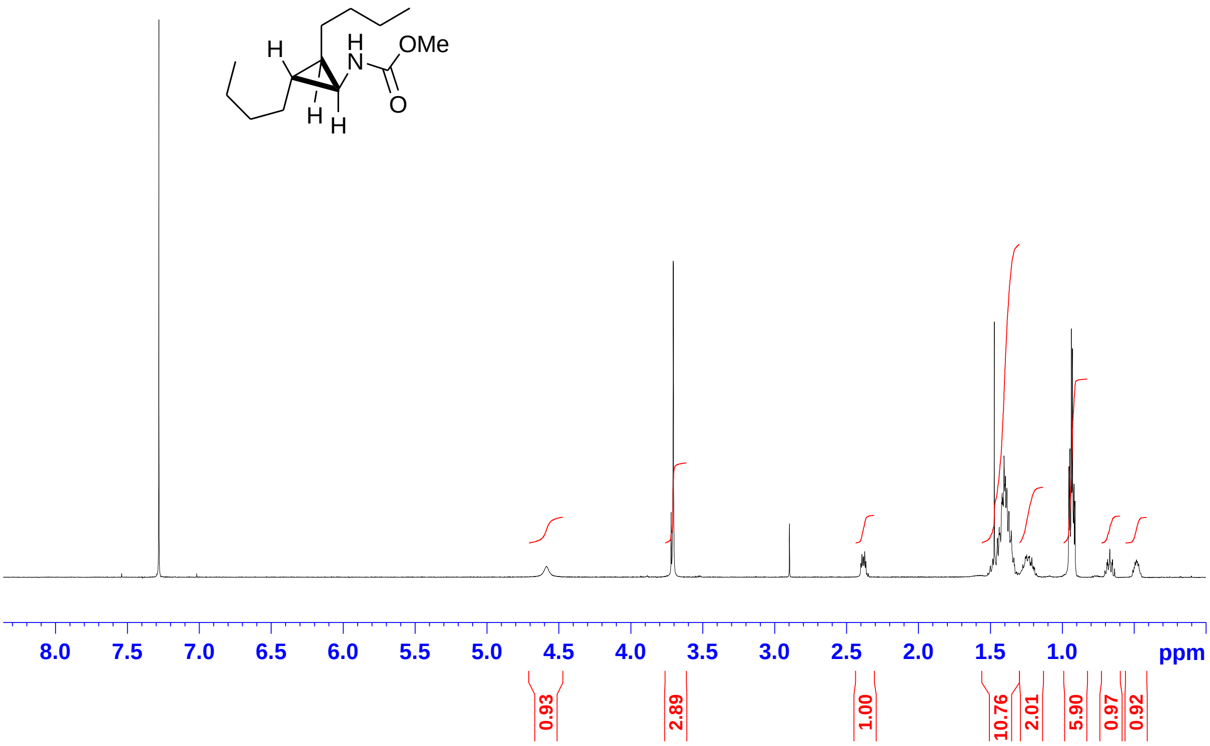
H
N
OMe
O
H
H H
8.0 7.5 7.0 6.5 6.0 5.5 5.0 4.5 4.0 3.5 3.0 2.5 2.0 1.5 1.0 ppm
0.93
2.89
1.00
10.76
2.01
5.90
0.97
0.92

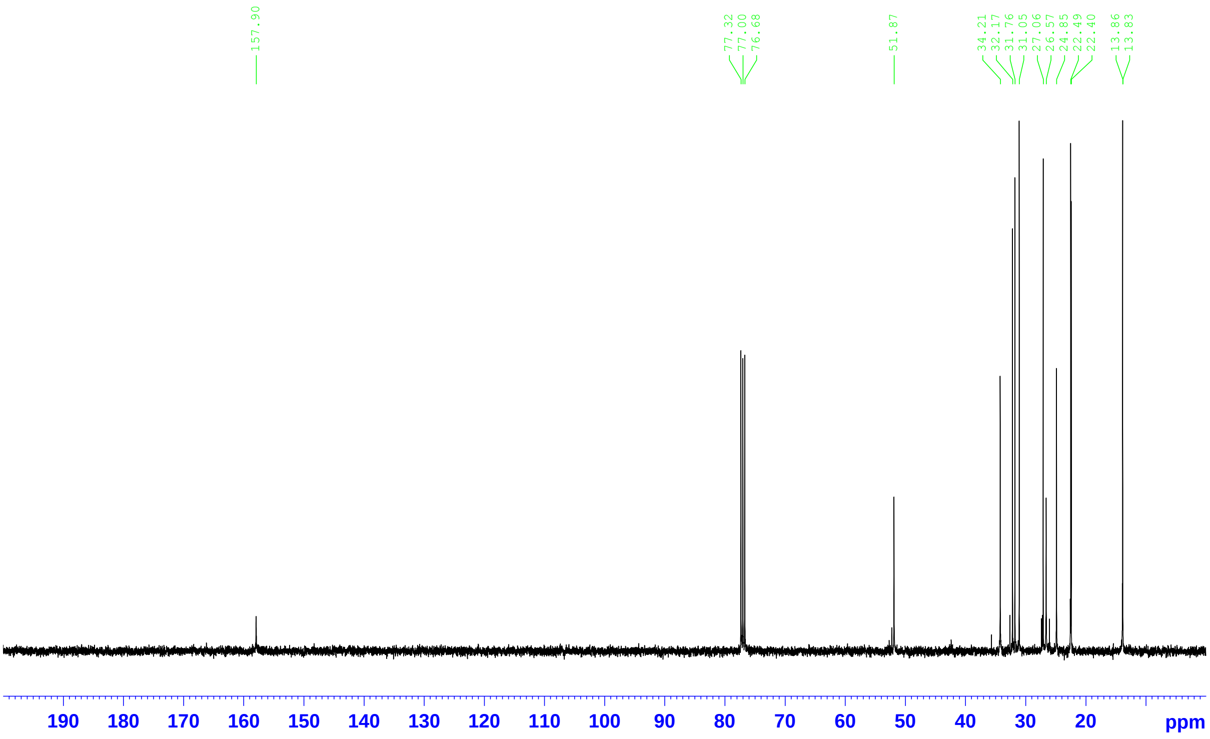
157.90
77.32
77.00
76.68
51.87
34.21
32.17
31.76
31.05
27.06
26.57
24.85
22.49
22.40
13.86
13.83
190 180 170 160 150 140 130 120 110 100 90 80 70 60 50 40 30 20 ppm

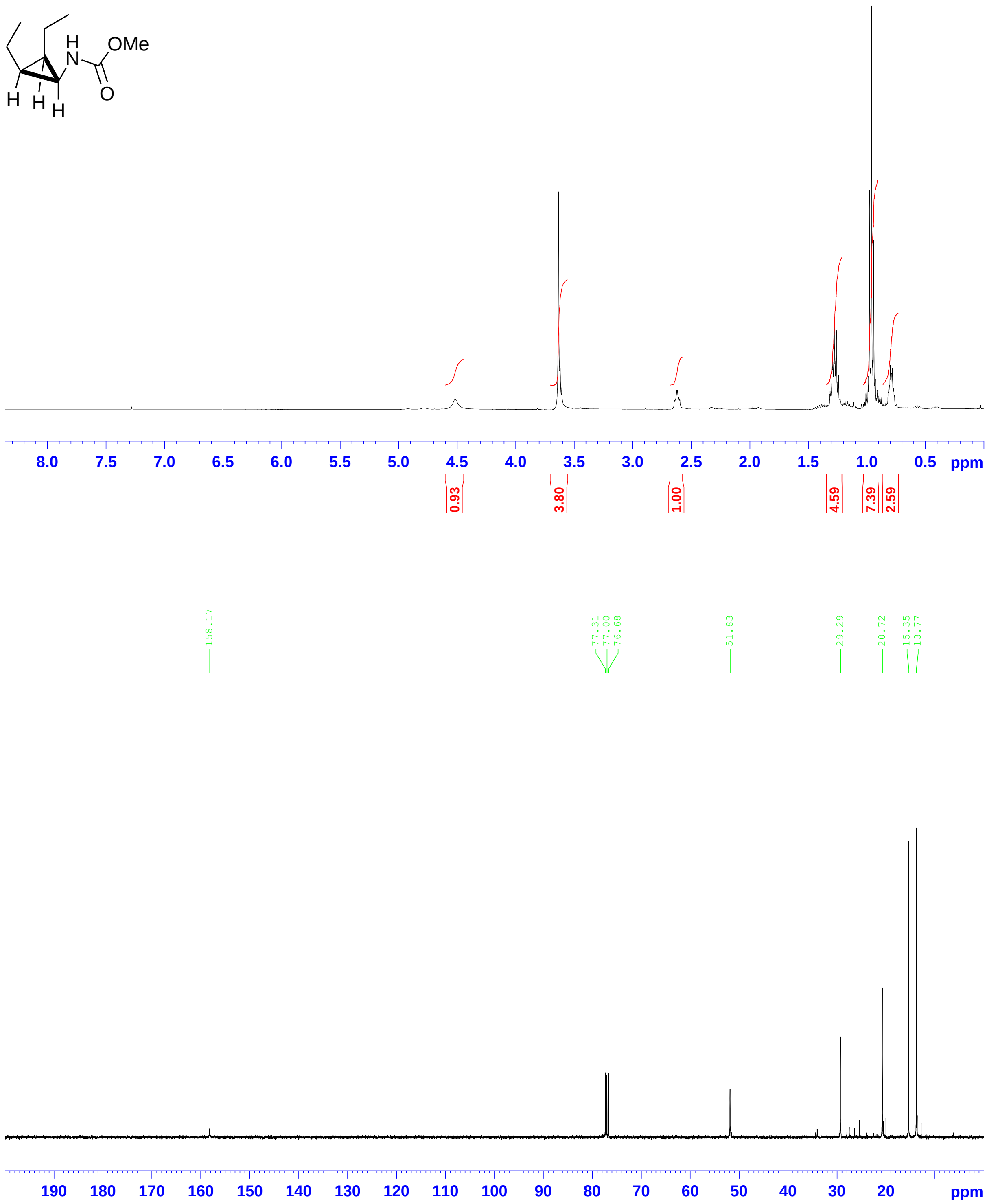
OMe
H
N
O
H H H
0.93
3.80
1.00
4.59
7.39
2.59
ppm
158.17
77.31
77.00
76.68
51.83
29.29
20.72
15.35
13.77
ppm

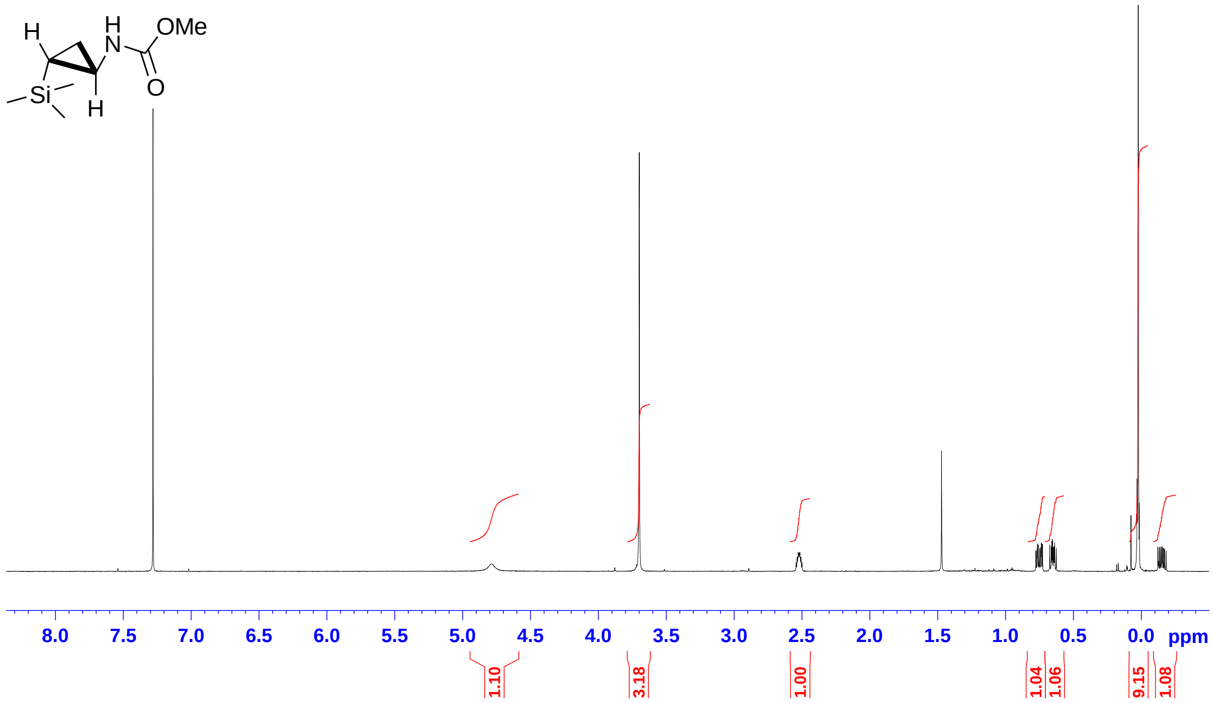
H
N
OMe
O
Si
H
H
8.0 7.5 7.0 6.5 6.0 5.5 5.0 4.5 4.0 3.5 3.0 2.5 2.0 1.5 1.0 0.5 0.0 ppm
1.10
3.18
1.00
1.04
1.06
9.15
1.08

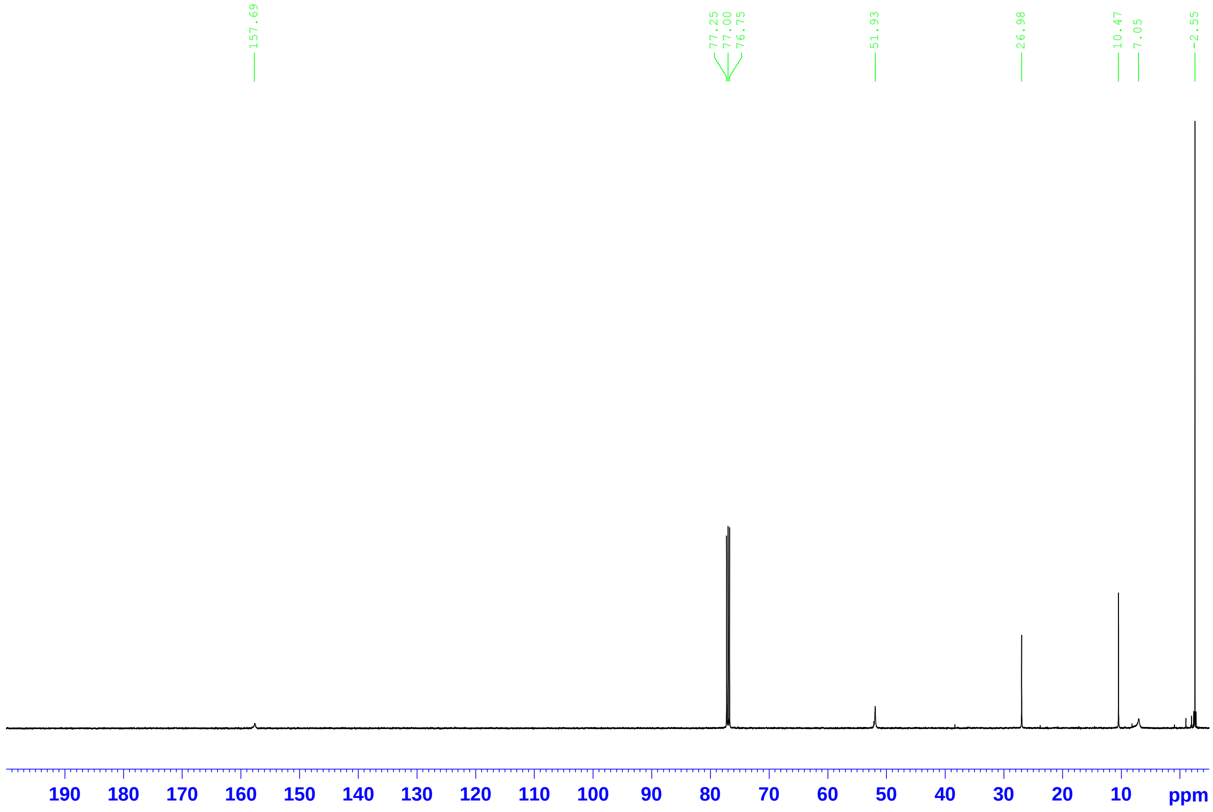
157.69
77.25
77.00
76.75
51.93
26.98
10.47
7.05
-2.55
190 180 170 160 150 140 130 120 110 100 90 80 70 60 50 40 30 20 10 ppm

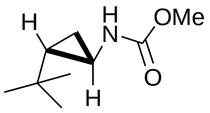
H
H
N
OMe
O
H

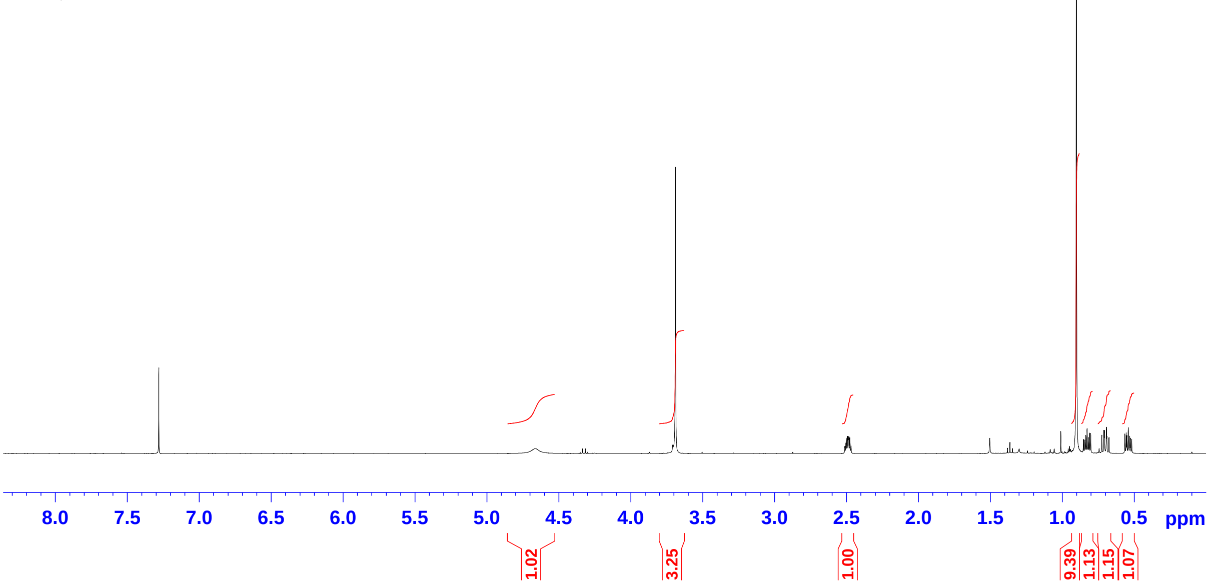
8.0 7.5 7.0 6.5 6.0 5.5 5.0 4.5 4.0 3.5 3.0 2.5 2.0 1.5 1.0 0.5 ppm
1.02
3.25
1.00
9.39
1.13
1.15
1.07

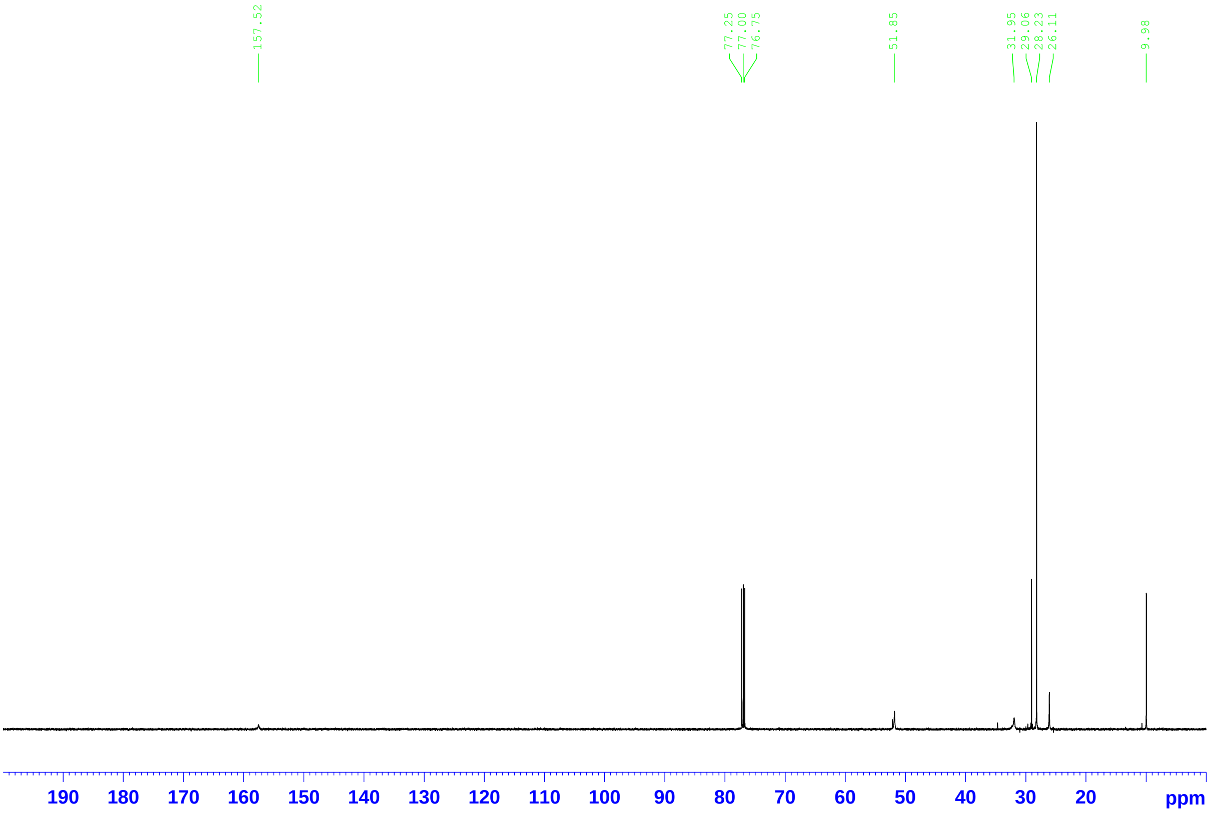
157.52
77.25
77.00
76.75
51.85
31.95
29.06
28.23
26.11
9.98
190 180 170 160 150 140 130 120 110 100 90 80 70 60 50 40 30 20 ppm

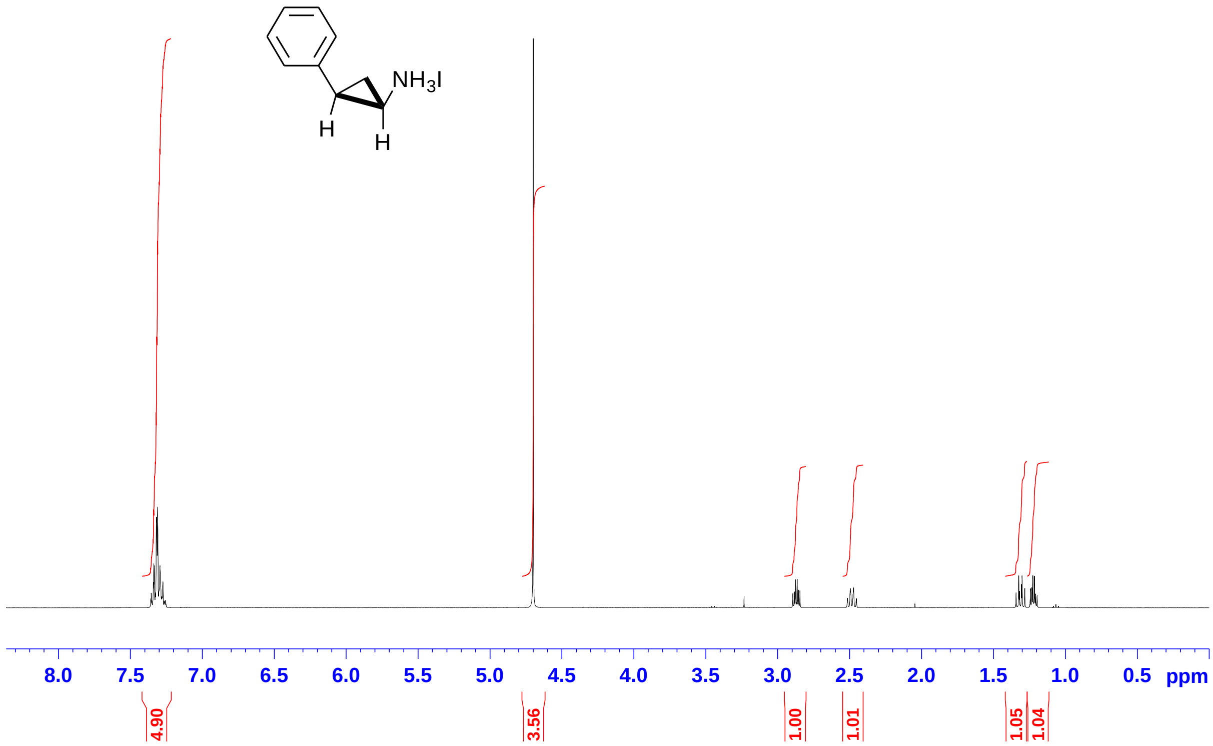
NH3I
H
H
4.90
3.56
1.00
1.01
1.05
1.04
8.0 7.5 7.0 6.5 6.0 5.5 5.0 4.5 4.0 3.5 3.0 2.5 2.0 1.5 1.0 0.5 ppm

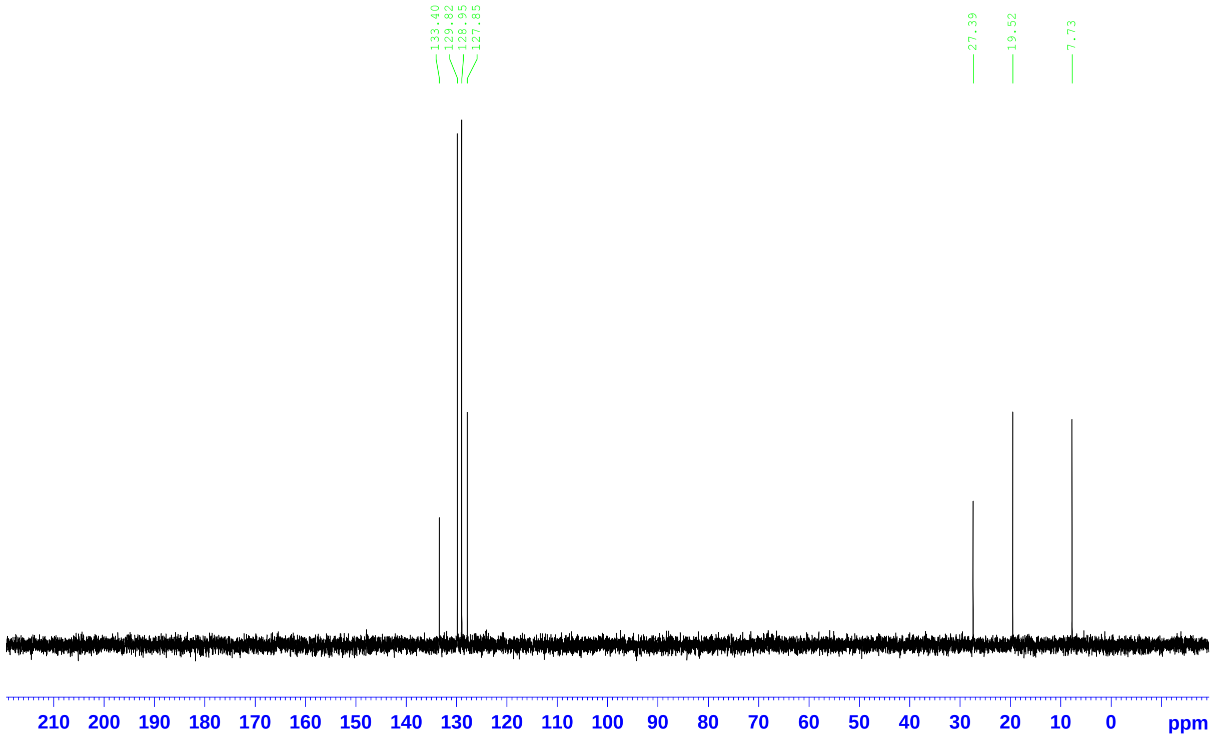
133.40
129.82
128.95
127.85
27.39
19.52
7.73
210 200 190 180 170 160 150 140 130 120 110 100 90 80 70 60 50 40 30 20 10 0 ppm

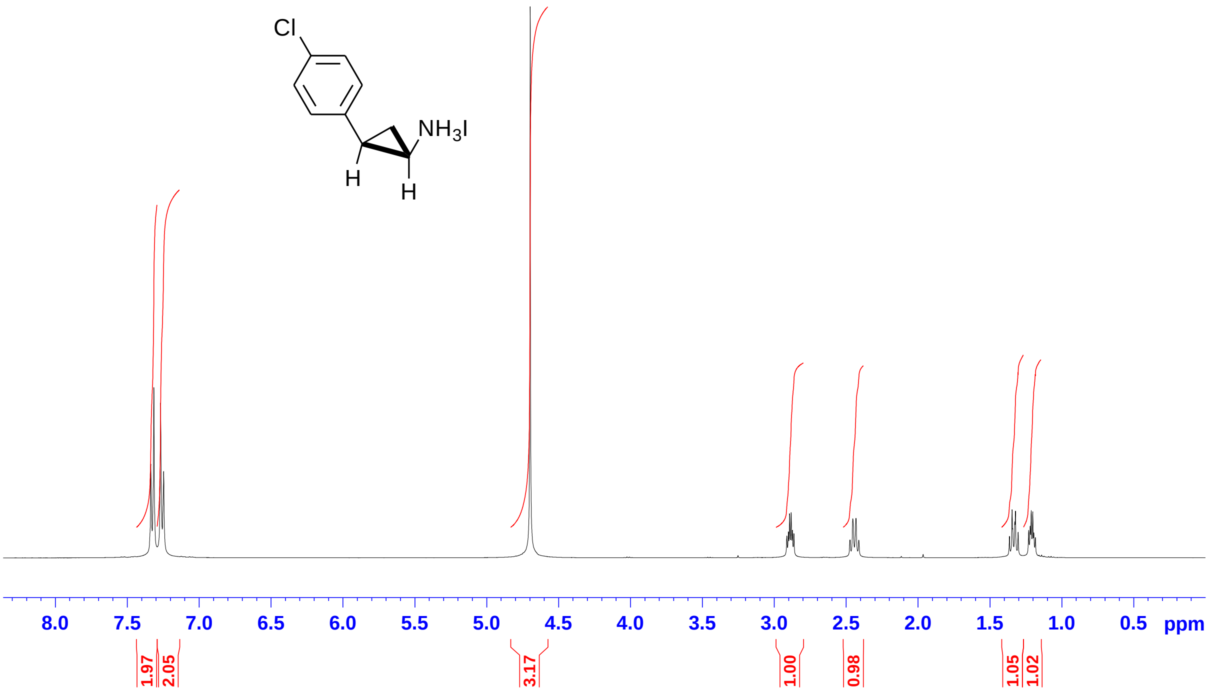
Cl
NH3I
H
H
8.0 7.5 7.0 6.5 6.0 5.5 5.0 4.5 4.0 3.5 3.0 2.5 2.0 1.5 1.0 0.5 ppm
1.97
2.05
3.17
1.00
0.98
1.05
1.02

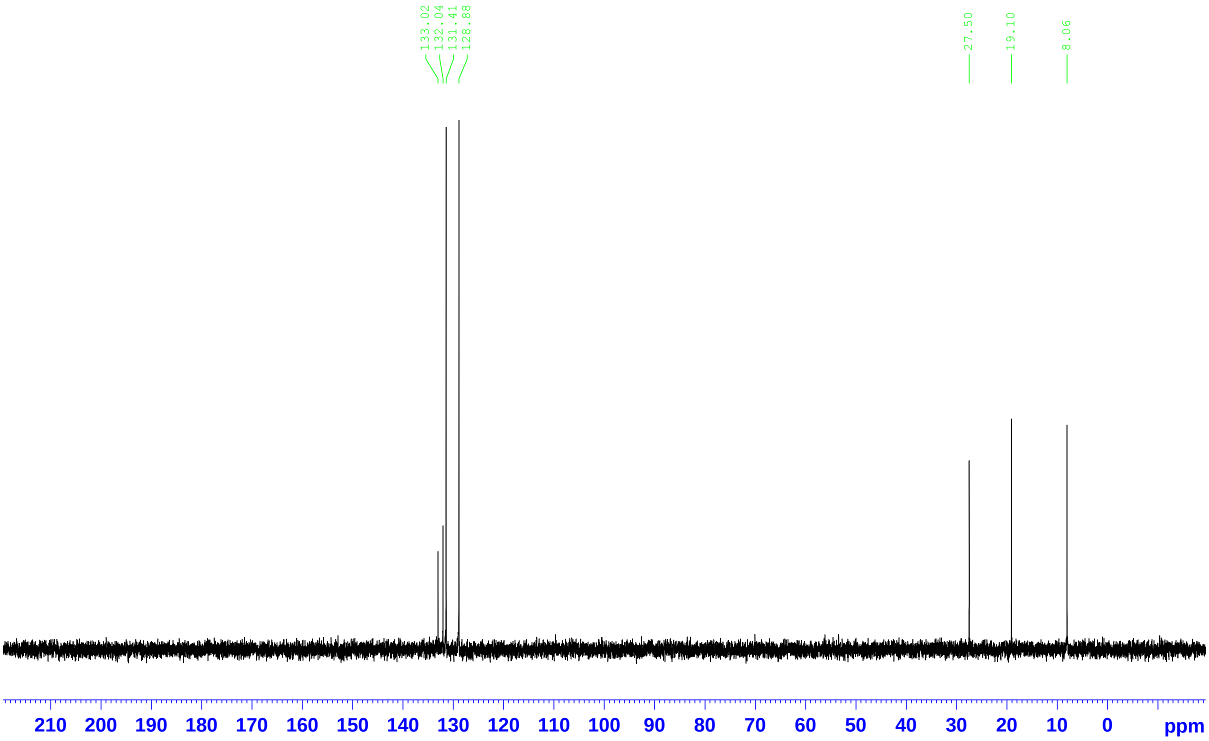
133.02
132.04
131.41
128.88
27.50
19.10
8.06
210 200 190 180 170 160 150 140 130 120 110 100 90 80 70 60 50 40 30 20 10 0 ppm

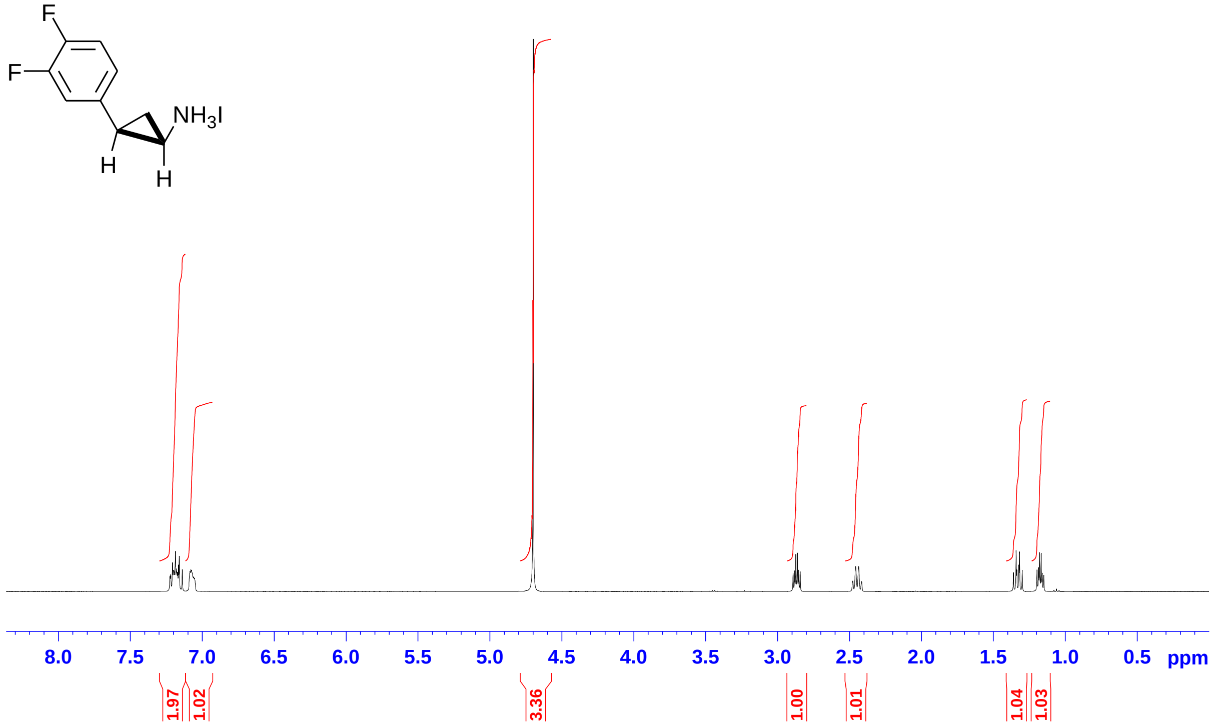
F
F
NH3I
H
H
8.0 7.5 7.0 6.5 6.0 5.5 5.0 4.5 4.0 3.5 3.0 2.5 2.0 1.5 1.0 0.5 ppm
1.97
1.02
3.36
1.00
1.01
1.04
1.03

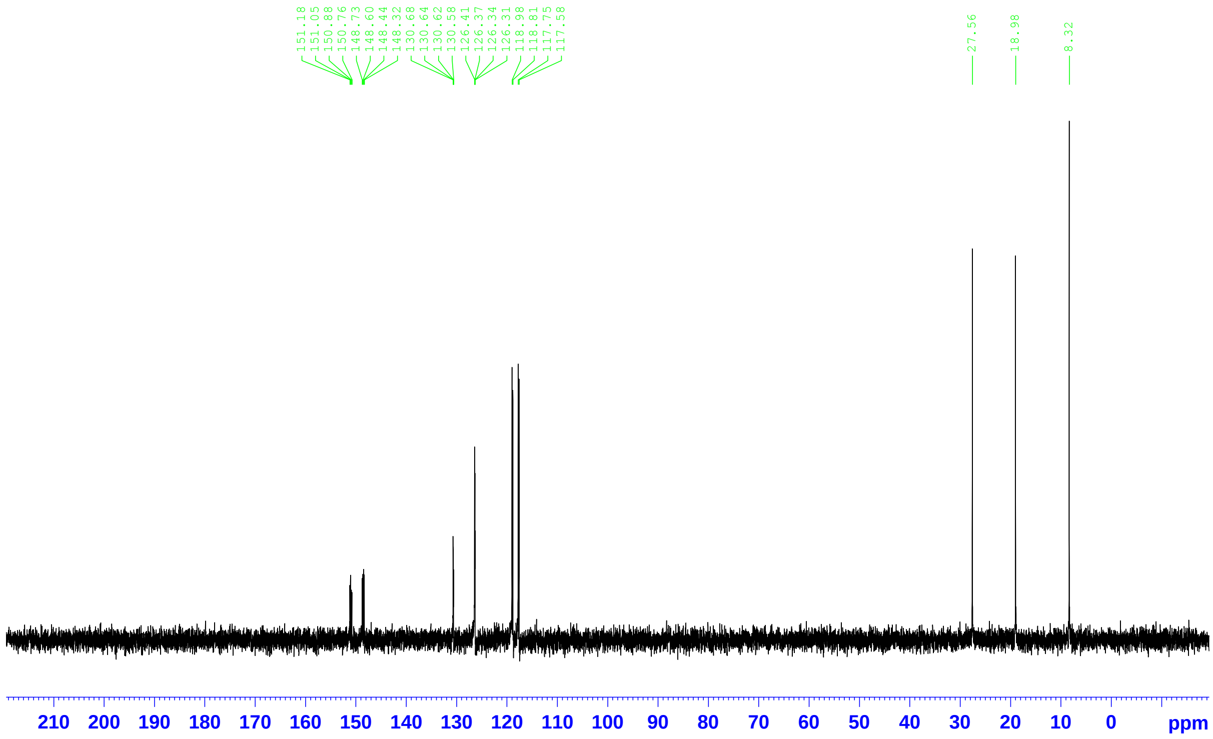
151.18
151.05
150.88
150.76
148.73
148.60
148.44
148.32
130.68
130.64
130.62
130.58
126.41
126.37
126.34
126.31
118.98
118.81
117.75
117.58
27.56
18.98
8.32
210 200 190 180 170 160 150 140 130 120 110 100 90 80 70 60 50 40 30 20 10 0 ppm

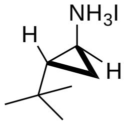
NH3I
H
H

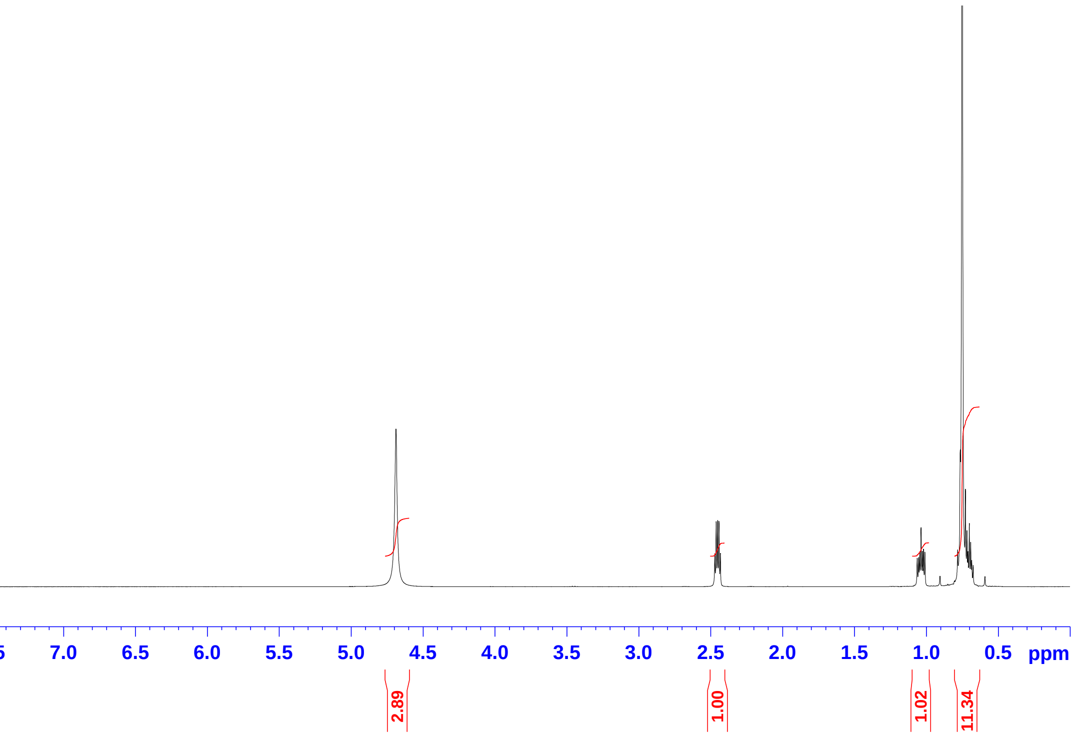
8.0 7.5 7.0 6.5 6.0 5.5 5.0 4.5 4.0 3.5 3.0 2.5 2.0 1.5 1.0 0.5 ppm
2.89
1.00
1.02
11.34

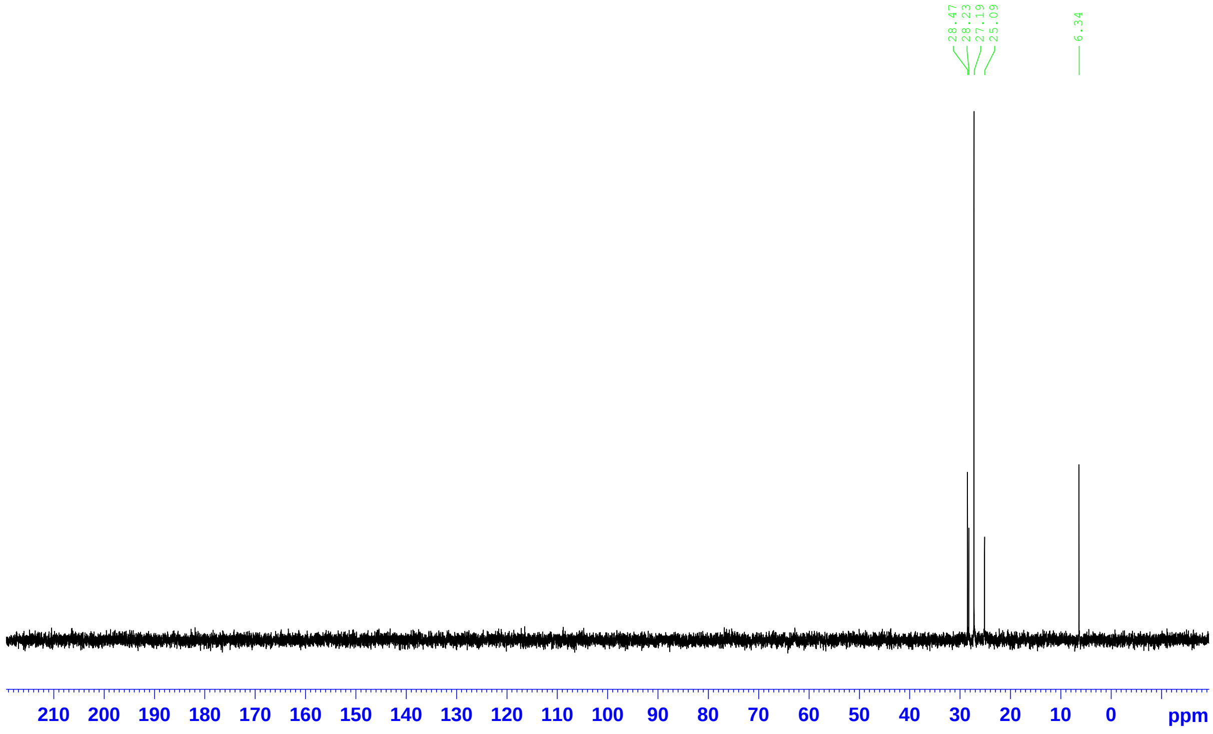
28.47
28.23
27.19
25.09
6.34
210 200 190 180 170 160 150 140 130 120 110 100 90 80 70 60 50 40 30 20 10 0 ppm

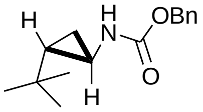
H
H
N
OBn
O
H

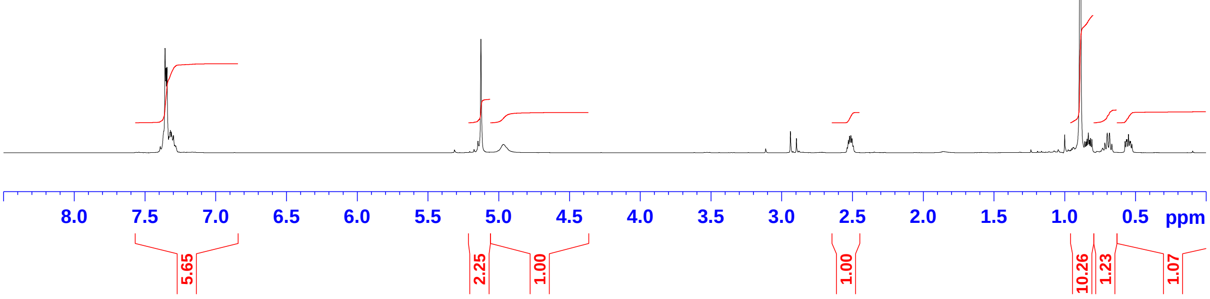
8.0
7.5
7.0
6.5
6.0
5.5
5.0
4.5
4.0
3.5
3.0
2.5
2.0
1.5
1.0
0.5
ppm
5.65
2.25
1.00
1.00
10.26
1.23
1.07

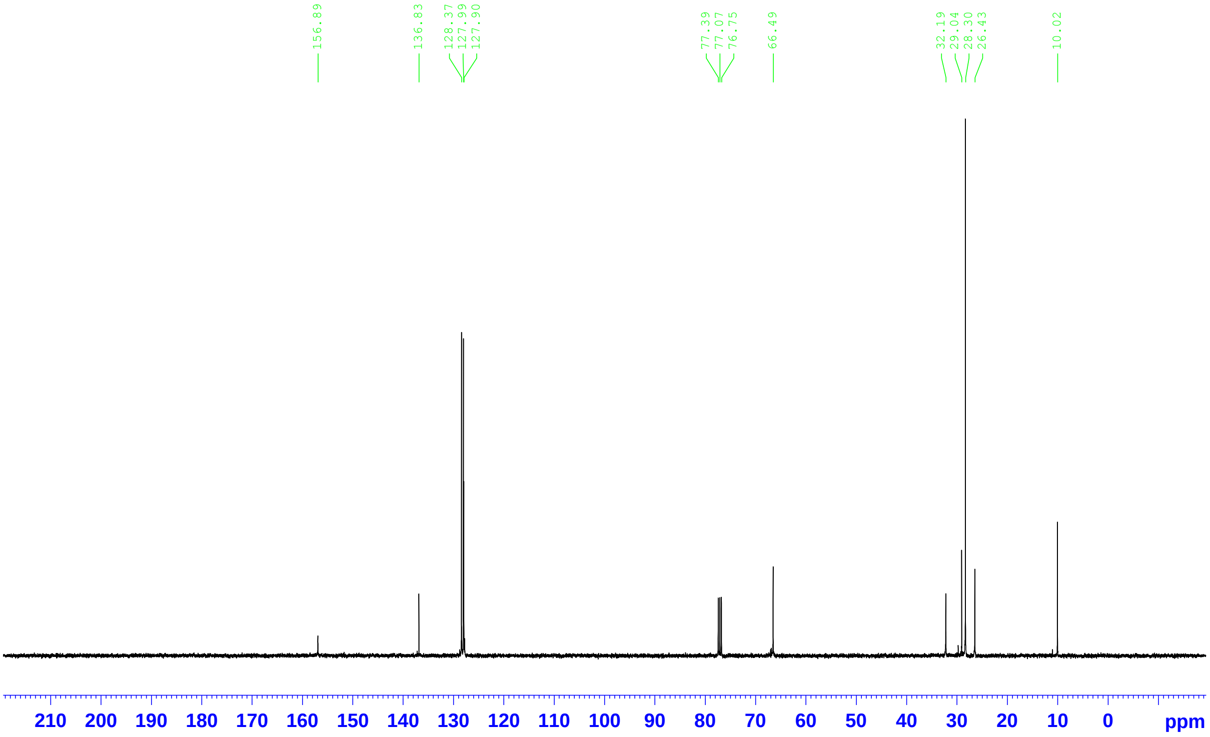
156.89
136.83
128.37
127.99
127.90
77.39
77.07
76.75
66.49
32.19
29.04
28.30
26.43
10.02
210
200
190
180
170
160
150
140
130
120
110
100
90
80
70
60
50
40
30
20
10
0
ppm
